# Integrating glycolysis, citric acid cycle, pentose phosphate pathway, and fatty acid beta-oxidation into a single computational model

**DOI:** 10.1038/s41598-023-41765-3

**Published:** 2023-09-02

**Authors:** Sylwester M. Kloska, Krzysztof Pałczyński, Tomasz Marciniak, Tomasz Talaśka, Beata J. Wysocki, Paul Davis, Tadeusz A. Wysocki

**Affiliations:** 1 Faculty of Medicine, Nicolaus Copernicus University Ludwik Rydygier Collegium Medicum, 85-094 Bydgoszcz, Poland; 2 Faculty of Telecommunications, Computer Science and Electrical Engineering, Bydgoszcz University of Science and Technology, 85-796 Bydgoszcz, Poland; 3 Department of Biology, University of Nebraska at Omaha, Omaha, NE 68182 USA; 4 Department of Electrical and Computer Engineering, University of Nebraska-Lincoln, Omaha, NE 68182 USA

**Keywords:** Carbohydrates, Systems biology, Biochemical networks, Bioenergetics, Computer modelling, Dynamic networks, Dynamical systems, Nonlinear dynamics, Numerical simulations, Stochastic modelling, Stochastic networks, Computational biology and bioinformatics, Biochemical reaction networks, Computational models, Data integration

## Abstract

The metabolic network of a living cell is highly intricate and involves complex interactions between various pathways. In this study, we propose a computational model that integrates glycolysis, the pentose phosphate pathway (PPP), the fatty acids beta-oxidation, and the tricarboxylic acid cycle (TCA cycle) using queueing theory. The model utilizes literature data on metabolite concentrations and enzyme kinetic constants to calculate the probabilities of individual reactions occurring on a microscopic scale, which can be viewed as the reaction rates on a macroscopic scale. However, it should be noted that the model has some limitations, including not accounting for all the reactions in which the metabolites are involved. Therefore, a genetic algorithm (GA) was used to estimate the impact of these external processes. Despite these limitations, our model achieved high accuracy and stability, providing real-time observation of changes in metabolite concentrations. This type of model can help in better understanding the mechanisms of biochemical reactions in cells, which can ultimately contribute to the prevention and treatment of aging, cancer, metabolic diseases, and neurodegenerative disorders.

## Introduction

Cellular metabolism modeling is an important but difficult task^1,2^. The difficulty arises from the fact that compounds, which act as substrates and products in the cell’s metabolic reactions, are like a system of interconnected vessels. Any change in the concentration of a compound in a cell indirectly affects other, seemingly unrelated compounds, and thus the reactions in which they participate. Many external as well as internal factors affect the course of reactions taking place in the cell, possibly accelerating, inhibiting, or blocking them. Due to the complexity of metabolism during computational modeling, it is necessary to adopt certain start and end points. Therefore, the best target for modeling seems to be those thoroughly studied metabolic pathways that are described in the scientific literature^3^. Nevertheless, it is necessary to determine certain simplifications that make such modeling possible. These simplifications may include the flow of metabolites between the cytoplasm and mitochondrion depending on the cell’s momentary demand, or the flow between different metabolic reactions, since the vast majority of metabolites are used in several different metabolic pathways.

Metabolic models can serve scientists in better planning of experiments. They allow predicting the effects of specific conditions on cell metabolism. Thanks to the ongoing development of metabolomics and computational biology, modeling can speed up the processes of diagnosing metabolic diseases and contribute to development of effective treatment methods^4^. In addition, suitably adapted models can map what happens in a cell under inhibition induced by a specific molecule or gene knockdown. It has long been known that cancer cells reprogram their metabolism and alter activity in pathways that are major sources of energy^5^. However, issues related to metabolism and cancer are still in the orbit of researchers’ interest^6,7^. In cancer, it has been found that many cancer cells have altered metabolism, often characterized by an increased reliance on glucose and a decreased reliance on oxygen for energy production. This metabolic reprogramming allows cancer cells to proliferate and survive in a hostile environment. Targeting the metabolic pathways of cancer cells is becoming a promising new avenue for cancer therapy. Recent research has shown that metabolism plays a key role in many fields that were previously not considered to be related to metabolism, such as aging^8^, and neurodegeneration^9,10^. In aging and neurodegeneration, it is becoming increasingly clear that metabolic dysfunction plays a key role in the development of these conditions. For example, research has shown that aging is associated with a decline in the function of the mitochondria, the cell’s power plants, which can lead to metabolic dysfunction. Similarly, many neurodegenerative diseases, such as Alzheimer’s disease, are associated with metabolic dysfunction in the brain. Targeting these metabolic pathways may be a promising new approach for treating these conditions. Overall, the emerging role of metabolism in these fields highlights the importance of understanding the complex interactions between metabolism and disease. Therefore, metabolic modeling and analysis can be used in cancer therapy, as it will contribute to testing the effects of specific molecules as early as at the planning stage of experiments. Another of the advantages of using metabolic models is that they reduce the number of laboratory animals used in research. Many times this type of research can lead to long-term damage to the health of these animals or even their death^11–13^.

The modeling method used in this work is based on queueing theory. Queueing theory is mainly used in issues related to engineering and telecommunications. However, there is evidence that it can be successfully used to model stochastic biological processes. Examples of applications of queueing theory to model biological processes include studies of signal transduction cascade in the cell^14^, insulin-related disorders and diseases^15^, glycolysis model^16^, tricarboxylic acid cycle (TCA cycle) model^17^, and the pentose phosphate pathway model^18^. The departure from deterministic models and the incorporation of fluctuations in metabolic simulations represent a significant advancement in our understanding of biological systems. Traditionally, deterministic models have been extensively used to study metabolic processes, assuming precise and predictable behavior. However, it is increasingly recognized that biological systems exhibit inherent stochasticity, where random fluctuations play a fundamental role in shaping cellular behavior. By implementing a flavor of the Kinetic Monte Carlo method^19^, similar to the Gillespie algorithm^20^, in our simulations, we have taken a crucial step towards capturing the effects of these fluctuations. Incorporating fluctuations in metabolic simulations is of utmost importance as it allows us to bridge the gap between the deterministic models and the real-world dynamics of biological systems. Fluctuations arise from various sources such as the discreteness of molecular species, spatial heterogeneity, and the inherent randomness of molecular interactions. Ignoring these fluctuations can lead to an incomplete and biased understanding of cellular processes.

By considering the inherent stochasticity in our model, we gain valuable insights into the behavior of metabolic networks that deterministic models fail to capture. Fluctuations have been shown to influence key aspects of cellular metabolism, including reaction rates, pathway efficiency, and robustness^21–23^. They can drive cellular decision-making, affect cellular responses to perturbations, and contribute to the emergence of complex phenomena at the system level.

Incorporating fluctuations in metabolic simulations also provides a more accurate representation of biological reality. By acknowledging the stochastic nature of cellular processes, we can better understand and reproduce experimental observations. Fluctuations play a role in generating the observed biological variability, and their inclusion in simulations allows us to better match experimental data and validate the model’s predictions. Moreover, by simulating fluctuations, we can explore the effects of different sources of variability, such as noise in gene expression or environmental fluctuations, on metabolic behavior. This information is crucial for understanding how cells respond and adapt to changing conditions and for unraveling the underlying principles governing cellular decision-making^24^.

It is important to note that incorporating fluctuations in metabolic simulations is not without challenges. Stochastic simulations can be computationally demanding, requiring specialized algorithms and efficient simulation techniques. However, advancements in computational power and the development of efficient algorithms, such as the Kinetic Monte Carlo method^19^, have made it increasingly feasible to simulate stochastic models at reasonable timescales. The departure from deterministic models and the incorporation of fluctuations in metabolic simulations represent a significant advancement in computational biology. By embracing the inherent stochasticity of biological systems, we gain deeper insights into the dynamics and behavior of metabolic networks, which would otherwise be overlooked by deterministic models^16^. Incorporating fluctuations allows us to better match experimental observations, understand biological variability, and explore the impact of stochasticity on cellular processes. These advancements pave the way for more accurate and comprehensive models of cellular metabolism and contribute to our overall understanding of complex biological systems.

The purpose of the present study was to develop an integrated computational model of the cell’s energy metabolism. This model consists of reactions included in important metabolic pathways and cycles, i.e. glycolysis, the pentose-phosphate pathway (PPP), the TCA cycle, and beta-oxidation. These are the pathways that play an important role in energy metabolism of the cell. Glycolysis is a simple metabolic pathway that regulates metabolic functions of various cells^25^, PPP is a pathway parallel to glycolysis, in which NADPH and 5-carbon sugars are generated^26^. Beta-oxidation is a series of reactions that break down long carbon chain fatty acids in order to generate acetyl-CoA and co-enzymes used in the electron transport chain, such as FADH$_{2}$ and NADH^27^. TCA cycle is an important metabolic pathway which uses acetyl-CoA produced in catabolic reactions of carbohydrate, fat, and protein metabolism, to generate energy^28^. TCA cycle is a source of various important biochemical compounds used in many other metabolic reactions in the cell. The presented model enables tracking of changes in the concentrations of individual metabolites of the aforementioned pathways. The innovation of this study is that the model has been based on queueing theory, compared to ODE-based models, which are commonly used for this kind of research. Another innovation is its nature that integrates pathways related to the formation and utilization of acetyl-CoA. In addition, it was showed that artificial intelligence algorithms can be successfully used to tune coefficients of the enzyme equations.

## Results

The TCA metabolites’ concentration values reported in the literature were either single-number measurements or ranges (Table 1). As a result, it was necessary to select not only the mean and standard deviation of the distribution but also the measurement values from ranges that maximize maximum log-likelihood estimation. This optimization problem was solved using GA.

**Table 1:** Statistical analysis of concentration values from literature.

Metabolite	Reported concentration $[$mmol/L$]$	Estimated mean	Estimated SD	Model conc. during glycolysis	Z-score
Acetyl-CoA	0.0288^29^	0.3022	0.2561	0.071	– 0.9028
	0.61^30^				
	0.07^2^				
	0.5^31^				
Oxaloacetate	0.00201^29^	0.0036	0.0010	0.005	1.4
	0.002–0.006^32^				
	0.005^2^				
Citrate	0.584^29^	0.6576	0.7103	0.184	– 0.8581
	2^30^				
	0.114^33^				
	0.4^2^				
	0.19^34^				
Isocitrate cis-aconitate	0.0321^29^	0.02604	0.0060	0.017	– 1.5067
	0.002–0.006^35^				
	0.02^31^				
$\alpha$-ketoglutarate	0.797^29^	0.5067	0.1973	0.031	– 2.411
	0.44^30^				
	0.25^2^				
	0.54^36^				
	0.004–0.013^37^				
Succinyl-CoA succinate	0.23^30^	0.2989	0.2710	0.720	1.5538
	0.0068^29^				
	0.66^36^				
	0.36–0.91^38^				
Fumarate	0.485^29^	0.6672	0.7496	0.488	– 0.2391
	0.12^30^				
	0.124^29^				
	1.94^36^				
Malate	1.7^30^	1.1137	0.4642	0.495	– 1.3328
	1.39^29^				
	0.495^36^				
	0.87^34^				

*SD* standard deviation.

The last column in the table presents the Z-score of modeled substrates’ concentration values regarding the estimated mean and standard deviation of the corresponding Gaussian distribution. All values of substrates, except for $\alpha$-ketoglutarate, have a Z-score between – 2 and 2. As a result, they are within two sigma distance from the estimated mean. Despite not being within the range of two sigmas for $\alpha$-ketoglutarate, our data still falls within the range of three sigmas.

During the experiment, glucose consumption in the cell was simulated. At the start of the model, the glucose concentration was fixed at 5 mM. This is a value in the range of normal blood glucose concentration^39^. In the initial phase of the simulation, the course of glycolysis, PPP, and TCA cycle reactions were modeled. The product of glycolysis, pyruvate, underwent reactions that converted pyruvate to oxaloacetate or acetyl-CoA, which are metabolites of the TCA cycle. Over the course of the simulation, the glucose concentration decreased. As the glucose concentration decreased, the reactions of the glycolysis pathway were extinguished. This was due to a decrease in the probability of occurrence of glycolysis reactions and, consequently, a decrease in the speed of these reactions. As a consequence of the decrease in glycolysis activity, the probability of occurrence of reactions entering the fatty acid beta-oxidation pathway increased, which, after glucose utilization, became the main source of acetyl-CoA used in the TCA cycle. The use of GA allowed combining the reaction of enzymatic kinetics of several energetically important biochemical pathways. Due to the large differences in numerical values between consecutive reactions, as well as influence of the reactions not included in the model on reaction rates, it was necessary to tune the model. GA proved to be an effective tool in this process.

**Table 2:** Comparison of concentration data between literature and model (mmol/L) at different time points of the simulation.

Metabolite	Conc. (literature) $[$mmol/L$]$	Model conc. at starting point	Model conc. during glycolysis	Model conc. during $\beta$-oxidation	Model conc. at the end of simulation	SD over mean
Acetyl-CoA	0.07^2^	0.070	0.071	0.060	0.060	0.002
Oxaloacetate	0.002–0.006^32^	0.006	0.005	0.001	0.001	0.126
Citrate	0.114^33^	0.190	0.184	0.115	0.110	0.031
Isocitrate cis-aconitate	0.002–0.006^35^	0.020	0.017	0.010	0.009	0.091
$\alpha$-ketoglutarate	0.004–0.013^37^	0.030	0.031	0.023	0.022	0.085
Succinyl-CoA succinate	0.36–0.91^38^	0.730	0.720	0.691	0.690	0.011
Fumarate	0.485^29^	0.485	0.488	0.490	0.490	0.012
Malate	0.495^36^	0.495	0.495	0.489	0.488	0.010

Comparison of concentration data between literature and model (mmol/L) at different time points of the simulation (20th second during glycolysis and 9000th second during beta-oxidation).

*SD* standard deviation.

**Figure 1:**
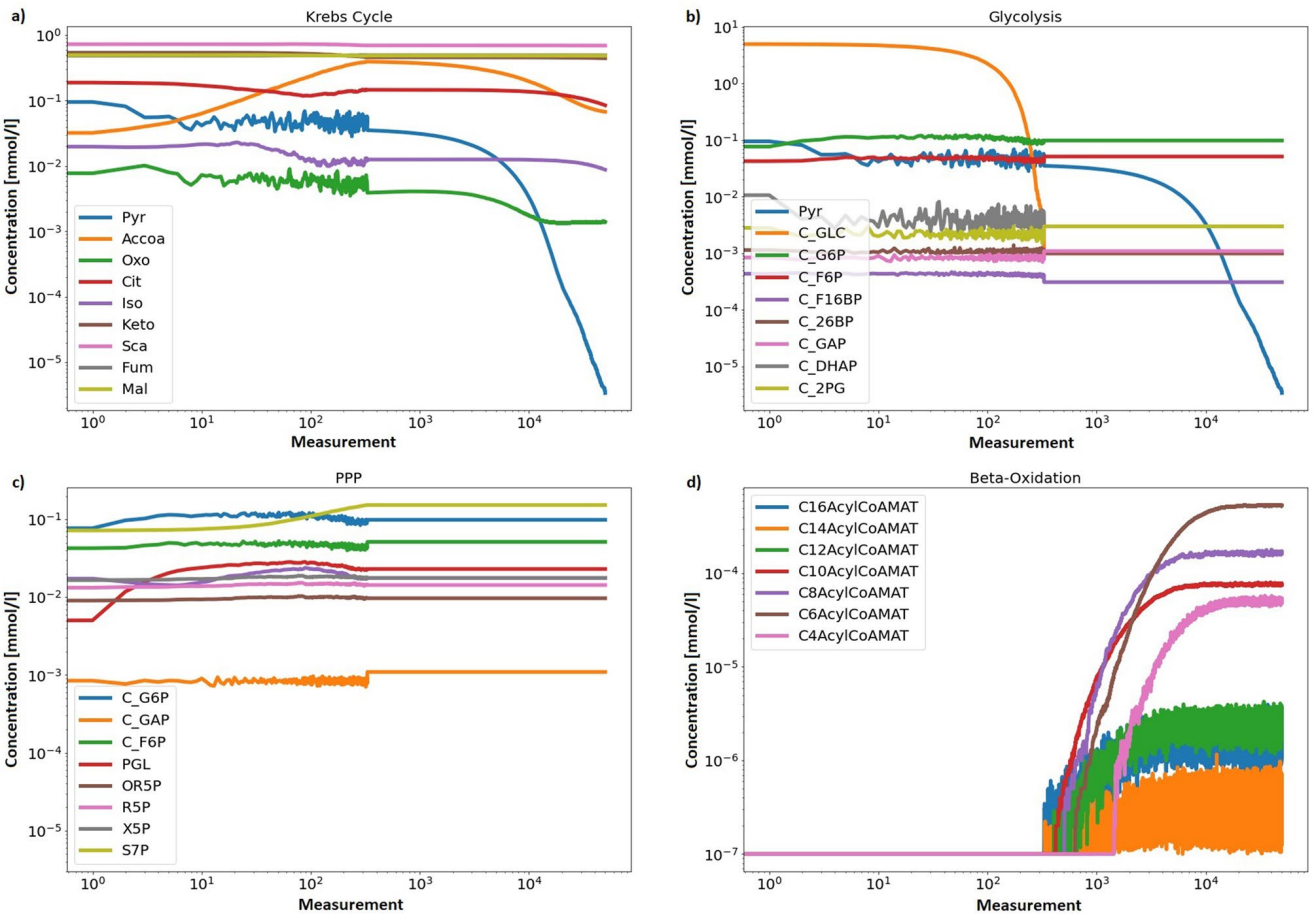
Visualization of the concentration change over the course of simulation in each of the modeled pathways: (**a**) TCA cycle, (**b**) Glycolysis, (**c**) pentose phosphate pathway, (**d**) beta-oxidation. The X-axis displays number of measurements. During 10,000 seconds of simulation, concentration was measured 50,000 times. The Y-axis displays the concentration of a given metabolite.

In order to check the validity of the results generated by the computational model, they were compared to concentration values measured under experimental conditions. For this purpose, the metabolites concentration values presented in scientific publications (Table 2) on the TCA cycle were used. The TCA cycle was chosen as a reference point due to the fact that it is a well-studied metabolic cycle and represents the final stage of the presented model. Table 2 presents the averaged results for 50 calculated cycles, mimicking 50 liver cells. The simulations covered a time interval of almost three hours (10,000 seconds). Each second, 5 measurements were taken, and their results were averaged and recorded. Changes in metabolite concentrations during the course of the simulations are presented in Fig. 1. The course of changes in the concentration of individual metabolites over time is stable. Compounds whose concentrations change the most over the course of the computational simulation, such as glucose and pyruvate, were expected to behave this way, since the model does not take into account glucose external replenishment over the course of the simulation.

The results presented in Table 2 indicate the high accuracy of the computed results with respect to the concentration values measured under laboratory conditions. The “SD over mean” column shown in Table 2 refers to the 90th percentile SD instead of the maximum SD due to the occurrence of outliers in the time series (e.g. sudden changes). In the case of oxaloacetate, the ”SD over mean” value is relatively high, due to the change in the inflow of this compound in the TCA cycle. Oxaloacetate in the initial phase of the simulation is supplied from two sources: (1) it is formed from pyruvate obtained in the glycolysis pathway and (2) it is formed from the acetyl-CoA conversion reaction. In the case of a longer simulation, as in the presented example, the first source related to glycolysis is extinguished, as the glucose concentration decreases, which is not kept constant in the presented model. In this model, the concentration of oxaloacetate in the long-term simulation is kept constant only by acetyl-CoA obtained by beta-oxidation of fatty acids. For the other TCA metabolites, the ”SD over mean” value is relatively low, relative to the value of the calculated concentration of these metabolites at specific time points. On this basis, it can be concluded that the model is stable, and the calculated concentration is not subject to sudden, high changes.

The observed discrepancies in the metabolite ranges compared to laboratory data are a significant aspect to address in our research. In order to develop our model, we relied on data obtained from diverse literature sources. It is important to acknowledge that the measurements reported in the literature exhibit considerable variability across different studies and sources. This variability arises from factors such as variations in laboratory setups, measurement techniques, experimental conditions, and potential inter-individual differences. It is crucial to recognize that the data we employed from the literature may not necessarily represent dynamic or steady-state biological measurements. Rather, these measurements often represent snapshots of metabolite concentrations taken under specific experimental conditions that may not precisely align with the steady-state conditions assumed in our model. Consequently, inherent discrepancies can arise between the measured values and the simulated results due to these variations in experimental setups. These factors highlight the need to carefully consider and address the limitations and sources of variability when interpreting and comparing our model outputs with laboratory data.

The results of the sensitivity analysis are presented in Table 3. The impact of the variance of acetyl-CoA and $\alpha$-ketoglutarate starting concentrations on substrates values at the end of each simulation was measured. It was decided to use these two metabolites as examples due to the fact that there are many various data on concentrations of these metabolites. In order to present sensitivity scores from dozens of different substrates obtained for various starting values of substances above, aggregation was used. Sensitivity scores from different substrates were concatenated into distributions and described by the distribution’s minimum, 5th percentile, median, 95th percentile, and maximum.

**Table 3:** Sensitivity analysis of impact generated by varying starting values of Acetyl-CoA and $\alpha$-ketoglutarate on end values of the substrates of each simulation.

Metabolite	Minimum	5th percentile	Median	95th percentile	Maximum
Acetyl-CoA	0.02	0.51	1.0	1.51	2.32
$\alpha$-ketoglutarate	0.24	0.66	1.0	1.64	13.23

The sensitivity scores were concatenated into the sensitivity distributions and described by their minimum, 5th percentile, median, 95th percentile, and maximum.

The change in activity of individual pathways, clearly depends on changes in glucose concentration. During the simulation run, the model strives to achieve the concentration values presented in the literature, while taking care to maintain the stability of the obtained results. We realize that the concentration of glucose in the cell under real conditions is maintained at a relatively constant level, such as through glycogenolysis or gluconeogenesis. However, due to the complexity and number of connections between biologically active molecules, the presented model does not take into account the maintenance of glucose at a constant level. By designing the model in this way, a change in the activity of the pathways is highlighted, changing the source of acetyl-CoA used in the TCA cycle. The presented results indicate the stability of the system, which is dependent on the concentration of glucose in the cell. It is also reflected in the sensitivity analysis. For each measured substrate, 90 percent of scores landed in close proximity to 1.0, thus implying the model’s robustness on changes in the starting values.

## Discussion

This paper introduces a comprehensive model of interconnected metabolic pathways, utilizing queueing theory, with the added benefit of being able to conduct real-time calculations that are not excessively complex. The experiment can be run on a regular desktop computer, as it does not require significant computing power. By examining glucose as a case study, the study illustrated that following carbohydrate depletion, the cell shifts its metabolic activity towards alternative sources of cellular energy, such as beta-oxidation of fatty acids (and potentially protein catabolism). These sources provide the necessary acetyl-CoA for energy conversion in the TCA cycle.

In our analysis, it is important to acknowledge that while the majority of TCA metabolites exhibited Z-scores within two standard deviations of the estimated mean, $\alpha$-ketoglutarate deviated slightly from this trend. This deviation may be attributed to biological variability or measurement uncertainties associated with $\alpha$-ketoglutarate in specific experimental conditions. Future research could delve deeper into understanding the factors contributing to this observation and explore potential biological implications. Additionally, our study demonstrates the effectiveness of the GA approach in optimizing the selection of data from ranges and underscores its utility in handling complex datasets with non-uniform measurement representations. This methodology can be readily applied to similar challenges in metabolomics and bioinformatics to improve the precision and reliability of data analysis, facilitating more accurate interpretations of metabolic pathways and their regulatory mechanisms.

Metabolic modeling plays a crucial role in bridging the gap between theoretical understanding and practical implementations in the field of biology. The ultimate aim of metabolic modeling is to provide insights that can guide the development of effective therapeutic approaches for metabolic disorders. By simulating and analyzing the intricate metabolic pathways within cells, we can unravel the underlying mechanisms and identify potential targets for intervention.

In this context, the queueing methodology utilized in our study offers distinct advantages over traditional methods such as ODEs and FBA. While ODEs assume continuous and deterministic behavior, the queueing methodology embraces the discrete and stochastic nature of biochemical reactions, providing a more realistic representation of cellular processes. By capturing the inherent variability and fluctuations in metabolic networks, the queueing methodology allows for a deeper understanding of the dynamic behavior and robustness of biological systems.

One key advantage of the queueing methodology is its ability to account for queueing delays and waiting times, which are essential factors in cellular processes. These delays reflect the finite capacity of cellular resources and the time required for reactants to interact and traverse various metabolic steps. By considering queueing phenomena, our methodology enables the investigation of how delays impact metabolic fluxes, reaction rates, and overall system behavior. Additionally, the queueing methodology offers unique insights into emergent properties and system-level behaviors that are challenging to capture using other methods. The inherent stochasticity and variability incorporated through the queueing approach allow for the exploration of rare events, transient behaviors, and non-equilibrium phenomena. This capability is particularly relevant in studying metabolic diseases, where small perturbations or rare events can have significant consequences for cellular function and overall health. Furthermore, the queueing methodology facilitates real-time tracking of metabolite concentrations, enabling dynamic simulations that closely mirror the temporal aspects of cellular metabolism. This temporal resolution provides a more comprehensive understanding of metabolic changes and their implications for cellular function.

By highlighting these distinctive features of the queueing methodology, we emphasize its potential in generating insights that cannot be obtained through traditional approaches like ODEs and FBA. The utilization of queueing theory enriches the toolbox of metabolic modeling, expanding the possibilities for practical applications in therapeutic development, personalized medicine, and precision interventions for metabolic disorders.

Metabolic changes are observed in various diseases, including metabolic disorders such as diabetes and obesity, as well as in the aging process^40^. These conditions have garnered significant attention from researchers due to the rising prevalence of metabolic disorders and their impact on health^41,42^. While regulatory pathways typically maintain metabolite concentrations within narrow bounds^43^, individual metabolite levels can vary among individuals and deviate from established norms. An example of altered metabolism is found in cancer^44^, where a process known as metabolic reprogramming occurs. Cancer cells exhibit a shift in energy utilization, bypassing the citric acid cycle in mitochondria and relying heavily on glycolysis, followed by lactate fermentation in the cytosol^45^.

In neurodegenerative diseases like Alzheimer’s and Parkinson’s disease, mounting evidence suggests that mitochondrial dysfunction plays a pivotal role in disease development and progression^9^. Studies have demonstrated reduced activity of the citric acid cycle in the brains of affected individuals^46^. One potential therapeutic approach for neurodegenerative diseases involves targeting the mitochondria and the citric acid cycle to improve their function. This can be achieved through various strategies, including increasing the levels of citric acid cycle enzymes such as citrate synthase or utilizing drugs that target specific enzymes within the cycle. Conversely, another approach involves reducing citric acid cycle activity by inhibiting enzymes, such as isocitrate dehydrogenase, which can help mitigate the production of reactive oxygen species (ROS) within mitochondria and reduce associated cellular damage^47^. Although these approaches are experimental, they hold promise for slowing disease progression and potentially ameliorating symptoms of neurodegenerative diseases. It is important to note that further research is needed to fully comprehend the therapeutic benefits of targeting the citric acid cycle in these conditions.

To date, most scientific publications have focused on modeling macronutrient balance. These studies were focused on different dietary states, so-called intermediate fasting or semi-starvation^48^, or the impact of an unbalanced diet on the development of metabolism-related diseases^49^. The information they contain is extremely valuable and provides a better understanding of metabolic disorders. The purpose of our work, however, was to focus on the changes in metabolism in relation to glucose concentration at the cellular level. By combining these different types of studies, a more comprehensive understanding of metabolism and related processes such as aging can be achieved. Computational modeling of metabolic pathways also holds the potential to expedite the development of effective therapeutic approaches for alleviating metabolic disorders.

There are several limitations to the presented model. Although it is a complex model that includes 68 reactions, it does not take into account numerous other reactions in which the metabolites are involved. The impact of these unaccounted reactions was evaluated using GA (see "Materials and methods"). Another limitation is that the accuracy of the model is dependent on the literature concentrations of metabolites and the kinetic parameters utilized in the model. Therefore, the model is subject to errors that may have arisen during the determination of concentrations and other parameters, such as K$_{M}$, K$_{i}$, and V$_{max}$ under laboratory conditions. We acknowledged this issue early on in the experiment and recognized that previously published data is something researchers must rely on and trust for the honesty of published outcomes. Consequently, we decided to use literature concentration values as initial values and compare simulation results to these values to evaluate the model’s accuracy. It should also be noted that, while the model results are consistent with literature values, we only observe the end results. This approach has the potential to accumulate errors in the middle phase of the experiment, leading to incorrect outcomes. However, the model’s stability, as illustrated in the results section, is in agreement with prior studies on the different pathways incorporated in the model^16–18^, thus reducing the likelihood of the aforementioned scenario. The presented outcomes demonstrate that the model is useful and appropriate for simulations of alterations in metabolite concentrations with high precision. In the future, we plan to refine the model and continue this research with the objective of creating an application that allows users to input their measured parameters and receive simulation outcomes for the entered values.

Our model is intentionally designed to be generic, incorporating data from various sources, tissues, and organisms due to the limitations in obtaining comprehensive and tissue-specific data from a single organism. However, we recognize the importance of tissue or cell type-specific applications in addressing specific biological questions. The modularity and flexibility of our model allow for the integration of tissue or cell type-specific data in future studies, which can enhance the relevance and applicability of our model to specific biological systems. By leveraging the power of queueing theory in conjunction with more precise and targeted data, we can achieve improved accuracy and gain deeper insights into tissue-specific metabolic dynamics. While our current study focuses on the broader implications of metabolic modeling and the advantages of queueing theory, we appreciate the reviewer’s comment as it highlights an important direction for future research, which can further enhance the biological relevance and applicability of our model.

## Methods

### Queueing theory

While ordinary differential equations (ODEs) have been widely used in computational modeling of biological processes, there are several factors to consider that suggest they may not be the ideal method for biological simulations. One important limitation is that ODEs are deterministic in nature, failing to accurately capture the inherent stochasticity often observed in biological systems. These systems exhibit discrete and random molecular interactions, which are better represented by stochastic simulation methods such as the Gillespie algorithm or agent-based modeling. In addition, negative results can occur in the course of calculations, requiring the use of non-negative ODE solvers^50^ such as in MATLAB. Furthermore, ODE models assume well-mixed conditions and neglect the spatial organization and heterogeneity commonly found in biological systems. However, spatial effects can significantly impact the dynamics of biochemical reactions. Alternative simulation methods, such as partial differential equations (PDEs) or spatial stochastic simulations, take into account the spatial aspects and may yield more accurate results for certain biological phenomena. In addition, ODE models heavily rely on precise knowledge of model parameters, including reaction rate constants and initial conditions. Yet, in many biological systems, these parameters are uncertain and can vary across individuals or experimental conditions. The presence of parameter uncertainty introduces variability and can affect the accuracy of ODE simulations. Alternative approaches like Bayesian inference or sensitivity analysis can help address parameter uncertainty and provide more robust predictions. Another consideration is the computational efficiency of ODE simulations. As mentioned earlier, ODEs can accumulate errors and become computationally demanding, especially for large-scale models or long simulation times. This computational burden restricts the exploration of complex biological systems or extensive parameter sweeps. To overcome these limitations, approximate or alternative simulation methods such as network-free methods or reduced-order modeling can offer more computationally efficient alternatives while still capturing essential dynamics. Moreover, certain biological systems exhibit emergent phenomena, which arise from collective interactions at the system level rather than being solely determined by individual molecular components. ODE models, focusing on the behavior of individual components, may fail to accurately capture these emergent properties. Other modeling techniques such as network models, agent-based modeling, or machine learning approaches can better capture these emergent behaviors and complex system-level dynamics. Considering these factors can provide researchers with a more comprehensive understanding of the limitations of ODEs in biological simulations. Exploring alternative modeling approaches that better suit the specific characteristics of the biological system under investigation will contribute to more accurate and insightful simulations.

Another approach used in the computational biology studies is flux balance analysis (FBA). FBA is a computational method used to study and analyze the metabolic capabilities of biological systems, particularly metabolic networks^51,52^. By assuming a steady-state condition, where the rates of production and consumption of metabolites within the network are balanced, FBA optimizes an objective function, typically biomass production, while considering mass balance and reaction constraints.

FBA offers several advantages in computational biology studies. Firstly, it demonstrates predictive power by computing the optimal flux distribution that maximizes the production of a specific metabolite or biomass. This enables researchers to make inferences about the metabolic capabilities of an organism under different conditions. Furthermore, FBA is suitable for high-throughput analysis, as it can handle large-scale metabolic networks. It can explore the behavior of thousands of reactions simultaneously, providing a comprehensive understanding of cellular metabolism. This makes it particularly useful for analyzing genome-scale metabolic models and conducting extensive studies. The constraint-based framework utilized by FBA simplifies the representation of complex biochemical networks. By relying on stoichiometric constraints, thermodynamic constraints, and steady-state assumptions, FBA becomes computationally efficient and mathematically tractable. This allows researchers to model and analyze metabolic networks in a practical manner^53^. However, FBA does have certain limitations. It assumes a steady-state condition, disregarding the temporal dynamics of metabolic networks. This means it cannot capture transient behavior or time-dependent responses of biochemical reactions, limiting its applicability in certain biological processes.

Moreover, FBA relies on several simplifying assumptions that may not hold true in all biological contexts. For instance, it assumes the absence of regulatory mechanisms and optimality of growth. These assumptions can limit the ability of FBA to capture the full complexity of cellular processes and may lead to deviations from real-world observations. Additionally, the accuracy of FBA predictions heavily relies on the completeness and accuracy of the metabolic network model used. Our knowledge of metabolic networks is still incomplete, and the absence of certain reactions or pathways in the model can affect the accuracy of FBA predictions.

In summary, FBA is a powerful computational tool for analyzing metabolic networks. It offers predictive capabilities, high-throughput analysis, and integration with experimental data. However, researchers should consider FBA’s assumptions about steady-state conditions, simplified representations of cellular processes, and its inability to capture temporal dynamics. FBA finds extensive application in metabolic engineering, drug target identification, and understanding disease metabolism in computational biology studies.

However, it is important to acknowledge that the methods mentioned above are not without limitations, which prompted us to explore the application of a queueing theory-based approach. Biochemical reactions occur in living organisms in an orderly fashion, and for this reason queueing theory seems well suited for use in such simulation-computing studies. The optimized model has low computational complexity and it is possible to track changes in metabolite concentrations in real time. In addition, using queueing theory, the nature of the simulation is closer to reality, because there is no possibility for negative results to occur, just as in a cell, metabolites cannot reach negative concentrations. Thus, there is no need for artificially forcing non-negative solutions as is the case with ODEs.

**Figure 2:**
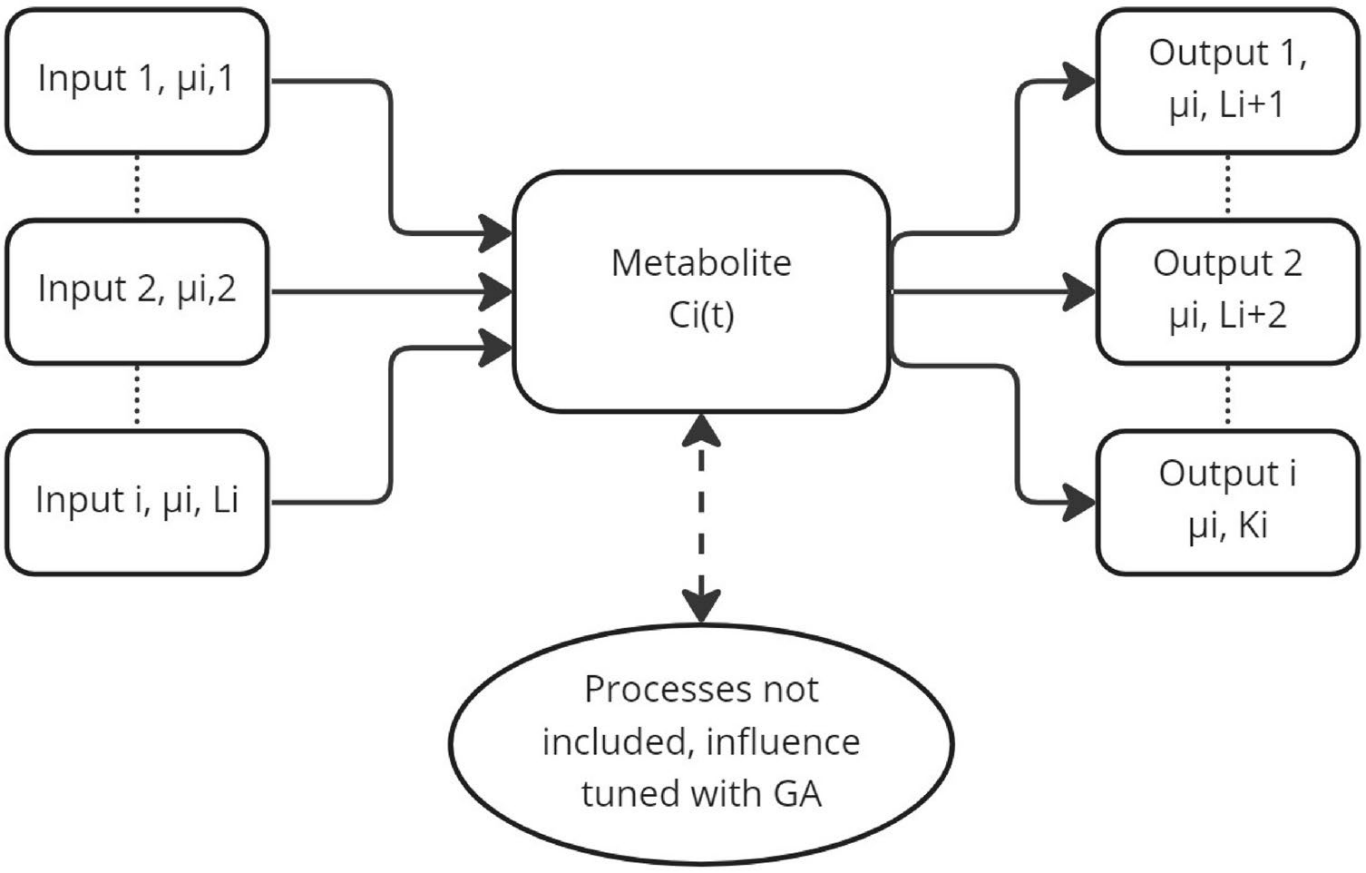
Example queue, which represents concentration Ci(t) of the metabolite. Arrival rates are presented as inputs, while metabolite depleting rates are outputs. Due to the complexity of the metabolic network, some simplifications were adopted (see more details in the “Enzyme kinetics” section). The influence of processes not included in the model were calculated using a genetic algorithm (GA).

The scheme of using queueing theory to model metabolite concentrations is shown in Fig. 2. The concentration of individual metabolites can be seen as a queue. Reactions affecting the increase in concentration of given queue are its inputs, while the reactions that consume the metabolite are its outputs. Processes affecting the concentration of a given metabolite that were not included in the enzyme kinetics equation were reduced to a factor determined using GA.

Various metabolic pathways, which are incorporated in the presented model can be mimicked by a composition of interconnected queues based on the Michaelis-Menten equations. The flow of metabolite concentration from one queue to another is sequential, so that a decrease in concentration in one queue will cause an increase in the next queue. Thus, a network of interrelated queues can be equivalent to a set of differential equations^54^.

The utilization of queueing theory as the foundation for our metabolic simulation model aims to capture the stochastic Markovian processes that represent variations in metabolite concentrations. To obtain the average change in concentration, we average the results from multiple simulation runs. At the core of this stochastic model are the Michaelis-Menten kinetic equations, which describe the relationship between substrate-product pairs and reaction velocities.

By representing a network of interconnected queues and digitizing the concentrations $C_{1}(t),...,C_{N}(t)$, we can effectively simulate the system. Within this modeling framework, the arrival rates function as queues, while the service rates correspond to the reaction rates $v_{(i,j(C_{1(t)},...,C_{N(t))},t))}$, normalized with respect to the simulation time step, $\Delta t_{i}$, and the concentration increment, $\Delta (C_{i}(t))$, reflecting the finite change of $C_{i}(t)$ within $\Delta t_{i}$.

It is important to note that we adopt a finite time increment, $\Delta t_{i}$, leading to a finite concentration increment, $\Delta (C_{i}(t))$. While this discretization introduces quantization error, adjusting the value of $\Delta (C_{i}(t))$ to minimize the error may entail increased computational time due to a reduced time step, $\Delta t_{i}$, necessitating more simulation steps to reach the desired duration. Thus, striking a balance is essential, and we calculate the normalization of reaction rates to achieve arrival and service rates for the queues using the given formula (Eq. 1).

1 $$\begin{aligned} \mu _{i,j} = \frac{|\upsilon _{i,j(C_{1}(t),...,C_{N}(t), t)}|\Delta t_{i}}{\Delta (C_{i}(t))} \end{aligned}$$

If the reaction rate $\upsilon _{i,j(C_{1}(t),...,C_{N}(t), t)}$ is positive, then its corresponding normalized rate, $\mu _{i,j}$, functions as an arrival rate. Conversely, if $\upsilon _{i,j(C_{1}(t),...,C_{N}(t), t)}$ is negative, the corresponding normalized rate, $\mu _{i,j}$, acts as a service rate. The instantaneous length of each queue embodies a potential realization of a stochastic Markovian process, capturing fluctuations in concentration for a specific metabolite. To obtain the average changes in concentration, we can compute the average of simulation results from multiple simulation runs.

To ensure the accuracy of the simulation, it is crucial to carefully select the simulation time step, $\Delta t_{i}$, and the concentration increment, $\Delta (C_{i}(t))$, such that all $\mu _{i,j}$ values are less than one. The arrival and service rates are representative of probabilities for the arrival and service of $\Delta (C_{i}(t))$ within the given time interval. To guarantee that a single $\Delta (C_{i}(t))$ is processed in each time interval, the following condition must hold (Eq. 2):

2 $$\begin{aligned} \mu _{i,j} \ll 1 \end{aligned}$$

for $$j = 1,..., K_{i}$$ and $$i = 1,..., N$$

However, it is not necessary for both the simulation time step, $\Delta t_{i}$, and the concentration increment, $\Delta (C_{i}(t))$, to be uniform across all $i = 1,..., N$. Instead, they can be chosen in a manner that minimizes simulation time while ensuring satisfaction of condition described in Eq. (2). Although dynamic calculation of time increments is feasible within each step, for the present model, we have opted for constant time increments for all reactions. This decision arises from the fact that some reaction rates differ significantly in orders of magnitude, making it impractical to utilize the shortest time increment that satisfies condition described in Eq. (2) for each reaction. By employing cumulative reaction time that remains uniform for all reactions, we can uphold the conservation of molar masses.

In recognition of the stochastic nature of chemical reactions, wherein reaction rates can vary under different environmental conditions, it is possible to introduce randomness by adding Gaussian (or other) noise to the kinetic constants used for computing values of $\upsilon _{i,j(C_{1}(t),...,C_{N}(t), t)}$. The same approach can be implemented at time instant, $t_{0}$, for the initial concentrations, $C_{(1)}(t_{0}),..., C_{(N)}(t_{0})$. This adaptation allows for a more realistic representation of the inherent fluctuations in chemical reactions, considering their sensitivity to environmental factors.

The reaction velocity serves as a macroscopic representation of numerous microscopic reactions, determining the frequency of reaction occurrence and its connection to the probabilities of increasing or decreasing specific substances. By utilizing these probabilities, we achieve a self-regulating and stochastic process that accurately simulates the behavior of biochemical pathways. The Michaelis-Menten kinetic equations calculate the probability of a reaction occurring based on substrate and product quantities, as well as kinetic constants and the duration of the time interval. These equations provide insights into the arrival and service rates in Poisson processes, where the arrival rate represents the probability of substance production, and the service rate represents the probability of substance consumption. The service time, which represents the interval between consecutive output events, is modeled using an exponential distribution. These assumptions align with classical queueing theory approaches, establishing a framework that integrates probabilities of increasing and decreasing substrates. This enables us to simulate biochemical pathways in a stochastic and self-regulating manner. In our model, the probability of a reaction occurring is determined by the Michaelis-Menten kinetic equations, where the concentration of metabolite-substrate and the kinetic constants play crucial roles. Each Michaelis-Menten equation is associated with a specific substrate and influences whether a reaction occurs at a given time point^16^. The reaction probability ranges from 0 to 1, and the reaction speed is considered a macroscopic representation of numerous microscopic reactions, resulting in the conversion of metabolites. The forward and reverse reaction velocities determine whether a metabolite increases or decreases. The probabilities of concentration gain and loss for each metabolite are correlated with the accumulation or increase in concentration of other metabolites. By adopting this approach, we have developed a self-regulating and stochastic model that integrates multiple metabolic pathways. The outcomes of the Michaelis-Menten equations can be interpreted as the arrival frequency and service rate in Poisson processes, with service times modeled using an exponential distribution (the time gaps between two consecutive output events). These suppositions align with traditional queueing theory methods. As a result, the count of arrivals in a specific time period $(t + \tau )$ follows a Poisson distribution with a parameter $\mu (t)\tau$ (Eq. 3):

3 $$\begin{aligned} P[(N(t + \tau )-N(t))=k, t]=\frac{e^{-\mu (t)\tau }(\mu (t)\tau )^{k}}{k!} \end{aligned}$$

where

${P[(N(t + \tau )-N(t))=k, t]}$ - probability of *k* arrivals in the interval (${{t, t + \tau }}$

$\mu (t)\tau$ - expected number of arrivals in a time interval duration of (${{t, t + \tau }}$]

The queue processing time of metabolite increment (Eq. 4) is described by the exponential distribution of the random variable *T* in the terms of the rate parameter $\mu (t)$.

4 $$\begin{aligned} f(T ; \mu (t))=\left\{ \begin{array}{ll} \mu (t) e^{-\mu (t) T} &{} T \ge 0 \\ 0 &{} T<0 \end{array}\right. \end{aligned}$$

Consequently, the arrival process at the beginning of the next queue, which the output of the examined server is linked to, follows a Poisson distribution. This is a complex stochastic process involving multiple variables, which are all connected to each other. As per the Michaelis-Menten kinetic equations, the likelihood of each packet arriving at a metabolite’s queue is linked to the quantity of product and inversely related to the quantity of substrate, leading to a self-regulating system that adjusts to the discrepancies of metabolites and ensures balance between arrivals and departures in every queue. One of the advantages of basing the model on queueing theory is the possibility for its further development and addition of more reactions/metabolic pathways without interfering with the previously optimized reactions. This is particularly interesting because the model can be developed with further metabolomics discoveries or combined with pathways not included in this study.

### Enzyme kinetics

The data used in the model for the values of metabolite concentrations and kinetic constants: K$_{M}$ (Michaelis constant), K$_{i}$ (inhibition constant), V$_{max}$ (maximum velocity), were obtained from scientific publications. It should be noted that these constants are not absolute values, but rely heavily on experimental conditions. In a seminal paper it was shown that modeling of yeast glycolysis requires actual redetermination of kinetic parameters under identical conditions for all enzymes^55^. However, the approach presented in this work aims to demonstrate a model that can be improved with further development of metabolomics, based on new, more accurate data supported by the application of GA. The collected data were used to describe metabolic reactions with Michaelis-Menten equations (Eq. 5) of enzyme kinetics. The model consisted of 68 enzymatic reactions of the form:

5 $$\begin{aligned} v(t)=\frac{V_{f}\frac{S_{1}(t)S_{2}(t)}{K_{S_{1}}K_{S_{2}}} - V_{r}\frac{P_{1}(t)P_{2}(t)}{K_{P_{1}}K_{P_{2}}}}{(1+\frac{S_{1}(t)}{K_{S_{1}}}+\frac{P_{1}(t)}{K_{P_{1}}})(1+\frac{S_{2}(t)}{K_{S_{2}}}+\frac{P_{2}(t)}{K_{P_{2}}})} \end{aligned}$$

where

$$\upsilon (t)$$ - reaction speed,

${V_{f}}$ - forward reaction speed,

${V_{r}}$ - reverse reaction speed,

${S_{1}(t), S_{2}(t),..., S_{x}(t)}$ - substrate concentration in mmol/L,

${P_{1}(t), P_{2}(t),..., P_{x}(t)}$ - substrate concentration in mmol/L,

${K_{S_{1}}, K_{S_{2}},..., K_{S_{x}}}$ - kinetic constant of substrate,

${K_{P_{1}}, K_{P_{2}},..., K_{P_{x}}}$ - kinetic constant of product.

It is assumed that all concentration values are sampled from Gaussian distribution specific to the type of examined concentration. The distributions were estimated using maximum log likelihood estimation^56^, given by the following equations (Eqs. 6 and 7):

6 $$\begin{aligned} \mu = \arg \max _{\mu } \sum _{i=1}^{n}\log (\frac{1}{\sqrt{2\pi \sigma ^{2}}}e^{-\frac{1}{2}(\frac{(x_{i}-\mu )}{(\sigma ^{2})})}) \end{aligned}$$

7 $$\begin{aligned} \sigma = \arg \max _{\sigma } \sum _{i=1}^{n}\log (\frac{1}{\sqrt{2\pi \sigma ^{2}}}e^{-\frac{1}{2}(\frac{(x_{i}-\mu )}{(\sigma ^{2})})}) \end{aligned}$$

Based on the kinetic equations, the probability of occurrence of each reaction was inferred, as described in the Materials and methods section related to Queueing theory. If the probability indicated that a reaction occurred, there was a decrease in the concentration of the metabolite that acted as a substrate in that reaction, while increasing the concentration of the metabolite that acted as a product. This is how the various reactions of the metabolic pathways included in the model gradually occurred, which were glycolysis, the pentose-phosphate pathway, the TCA cycle, and beta-oxidation. In case of missing literature data on reverse reaction speed, we applied the assumption of^57^ which describes a reverse reaction as 100x slower than the forward reaction. In few cases where the literature review did not provide enzyme kinetic data, the concentrations of two adjacent metabolites were summed and combined into a single metabolite (queue). In such a case, enzyme kinetics data on the second metabolite of the pair were used^17^. In the study presented here, such a situation occurred twice, when describing kinetic reactions involving isocitrate and cis-aconitate as well as succinyl-CoA and succinate, which are the metabolites of TCA cycle. To model such a complex metabolic network, it was necessary to establish a specific, rigid framework and scope of model coverage. The influence of cellular processes that are not directly included in the kinetic equations, such as the flow of a metabolite between compartments of a cell, was finetuned using a genetic algorithm (GA)^17^.

### Genetic algorithm

The equations were supplemented with coefficients selected using a GA. The choice of the algorithm was made arbitrarily, due to its effectiveness in previous similar studies that have been conducted^17,18^. This procedure was intended to allow combining the reactions of pathways whose computational values of individual reactions can differ markedly. The GA plays a crucial role in our study by optimizing the parameter values within the ranges reported in the literature. It is important to note that these parameter values can vary significantly between different studies and cell types. In order to achieve system stability and ensure consistency with experimental values, the GA searches for parameter values that allow the model to approximate the observed behavior. By employing the GA, we aim to find parameter values that not only make the system stable but also provide results that are consistent or approximate to experimental values. The algorithm iteratively explores the parameter space, evaluating different combinations of parameter values, and selecting those that best align with the experimental data.

The loss function was calculated with the use of ‘chromosomes’ that consist of two parameters: (1) metabolite’s concentration described in the literature and (2) a current optimization stage of the simulations. There are one hundred ’chromosomes’ in the population, each of which is a potential solution for the table of kinetic constants. Evaluation of the ‘chromosome’ involves using its ‘genes’ as the values of constants parametrizing Michaelis-Menten equations. In this process, the simulation time series is generated.

The resulting time series is sampled at fixed time stamps in order to compare simulated results with real-life experiments results registered in the literature. The loss function quantifying fitness of the ‘chromosomes’ is the sum of squares of the distances of the sampled points from simulation time series to the literature results (Eq. 8).

8 $$\begin{aligned} g_{p}: \hat{X}, X \rightarrow \sum _{i=1}^{|X|}\left( \frac{\hat{X}_{i}-X_{i}}{X_{i}}\right) ^{2} \end{aligned}$$

where

$g_{p}$ - subfunction that penalizes the difference between two vectors in relation to second vector,

*X* - vector of substrate concentrations described by a literature,

$\hat{X}$ - vector of substrate concentrations obtained by evaluation.

The loss function is designed to guide the GA to identify a ‘chromosome’ with a table of kinetic constants that leads to stable concentrations of products and minimizes the distance between initial values and stable points, which generates computational results that are closest to those obtained in laboratory measurements. Evaluating one ’chromosome’ entails running a simulation using its set of genes as the table of kinetic constants. The simulation function returns the values of substrate concentrations at each second. This table is used by the equation to determine the ’chromosome’s’ score. The function calculates the average vector of the last 100 recordings and computes the absolute difference with the initial simulation concentrations. In the final step, the average of the differences is calculated. The ’chromosome’ that minimizes this function is selected as the optimal table of kinetic constant values. The evaluation of each ’chromosome’ is done by simulating the model for the first hour. There are 100 ’chromosomes’ in the population at each step of optimization, and only the 10 sets of constants that minimize the fitness function are selected for reproduction. The reproductive algorithm is a variation of the standard crossover with an additional mechanism to prevent finding a trivial solution to minimize the loss function problem, which is to zero the probability of every reaction. The main disadvantage of the fitness function described above is the existence of a trivial solution for its minimization problem. If the ’chromosome’ contains only zeros, then no reaction would occur, so the settling points of concentrations of products in the model would have the same values as initial concentrations, thus finding a global minimum. To prevent the GA from converging to this solution, the reproduction mechanism requires that each reaction at $t=0$ has a probability of being performed between 1% and 10%. Reaction and balancing flow rates have ranges from 1 to 10% at the beginning of the simulation, which starts from substrate concentration values described in the literature. Applying these constraints to the reaction rates prevents them from being zeroed at the start and also prevents saturation of reactions. The reproduction algorithm has a 10% chance to perform a mutation with the mutation amplitude equal to 1.0. The optimization performed with GA was based on experimental measurements. The relative square error between subsequent values of the obtained vector and reference vector were used to calculate the penalty subfunction. To enforce equal contributions of all substrates in the optimization process, division by the value from the reference vector was performed.

### Sensitivity analysis

The resulting simulations of the trained model were subjected to the variance-based sensitivity analysis. It is used to analyze the sensitivity of a model’s output to changes in the input variables. It is based on the idea that the variance of the output of a model can be used to measure the model’s sensitivity to changes in the input variables (Eq. 9):

9 $$\begin{aligned} S_{j} = |\frac{V(E_{Y}|\mu _{j})}{V(Y)}| \end{aligned}$$

where

$E_{Y}$ - expected value of the signal Y,

*V*(*Y*) - variance of signal Y,

$V(E_{Y}|\mu _{j})$ - a variance of signal *Y* generated using input value *j*.

In this paper, *Y* represents a set of values of one substrate at the end of each simulation. Each value is a result of a simulation conducted using specific starting values denoted as *j*. The sensitivity analysis was conducted for each substrate making the cell’s measurable state.

### Supplementary Information

Supplementary Information.

## Data Availability

All data generated or analysed during this study are included in this published article and its supplementary information files. The datasets generated and/or analysed during the current study are available in the GitHub repository, https://github.com/UTP-WTIiE/CellEnergyMetabolismModel, DOI:10.5281/zenodo.7585089, implemented in C# supported in Linux or MS Windows.

## References

[1] Ederer M (2014). A mathematical model of metabolism and regulation provides a systems-level view of how Escherichia coli responds to oxygen. Front. Microbiol..

[2] Nazaret C, Heiske M, Thurley K, Mazat J-P (2009). Mitochondrial energetic metabolism: A simplified model of TCA cycle with ATP production. J. Theor. Biol..

[3] Becker SA, Palsson BO (2008). Context-specific metabolic networks are consistent with experiments. PLoS Comput. Biol..

[4] Mardinoglu A (2013). Integration of clinical data with a genome-scale metabolic model of the human adipocyte. Mol. Syst. Biol..

[5] Phan LM, Yeung S-CJ, Lee M-H (2014). Cancer metabolic reprogramming: Importance, main features, and potentials for precise targeted anti-cancer therapies. Cancer Biol. Med..

[6] Pal S, Sharma A, Mathew S, Jaganathan B (2022). Targeting cancer-specific metabolic pathways for developing novel cancer therapeutics. Front. Immunol..

[7] Perri F (2022). Cancer cell metabolism reprogramming and its potential implications on therapy in squamous cell carcinoma of the head and neck: A review. Cancers.

[8] Jang JY, Blum A, Liu J, Finkel T (2018). The role of mitochondria in aging. J. Clin. Investig..

[9] Han R, Liang J, Zhou B (2021). Glucose metabolic dysfunction in neurodegenerative diseases-new mechanistic insights and the potential of hypoxia as a prospective therapy targeting metabolic reprogramming. Int. J. Mol. Sci..

[10] Muddapu VR, Dharshini SAP, Chakravarthy VS, Gromiha MM (2020). Neurodegenerative diseases-is metabolic deficiency the root cause?. Front. Neurosci..

[11] Hajar R (2011). Animal testing and medicine. Heart Views Off. J. Gulf Heart Assoc..

[12] Hawkins P (2019). Avoiding Mortality in Animal Research and Testing.

[13] Lynch J, Slaughter B (2001). Recognizing animal suffering and death in medicine. West. J. Med..

[14] Tsuruyama T (2023). Kullback-Leibler divergence of an open-queuing network of a cell-signal-transduction cascade. Entropy.

[15] Uygulanması İ (2007). An application of queueing theory to the relationship between insulin level and number of insulin receptors. Türk Biyokimya Dergisi Turk. J. Biochem..

[16] Clement EJ (2020). Stochastic simulation of cellular metabolism. IEEE Access.

[17] Kloska S (2021). Queueing theory model of Krebs cycle. Bioinformatics.

[18] Kloska SM (2022). Queueing theory model of pentose phosphate pathway. Sci. Rep..

[19] Guang W (1998). Application of queueing theory with monte Carlo simulation to the study of the intake and adverse effects of ethanol. Alcohol Alcohol..

[20] Gillespie DT (1977). Exact stochastic simulation of coupled chemical reactions. J. Phys. Chem..

[21] Cheong R, Rhee A, Wang CJ, Nemenman I, Levchenko A (2011). Information transduction capacity of noisy biochemical signaling networks. Science.

[22] Kiviet DJ (2014). Stochasticity of metabolism and growth at the single-cell level. Nature.

[23] Selimkhanov J (2014). Accurate information transmission through dynamic biochemical signaling networks. Science.

[24] Kitano H (2002). Systems biology: A brief overview. Science.

[25] Guo X (2012). Glycolysis in the control of blood glucose homeostasis. Acta Pharm. Sin. B.

[26] Alfarouk KO (2020). The pentose phosphate pathway dynamics in cancer and its dependency on intracellular ph. Metabolites.

[27] Houten SM, Wanders RJ (2010). A general introduction to the biochemistry of mitochondrial fatty acid $$\beta $$-oxidation. J. Inherit. Metab. Dis..

[28] Ponizovskiy M (2016). Role of Krebs cycle in mechanism of stability internal medium and internal energy in an organism in norm and in mechanism of cancer pathology. Mod. Chem. Appl..

[29] Park JO (2016). Metabolite concentrations, fluxes and free energies imply efficient enzyme usage. Nat. Chem. Biol..

[30] Bennett BD (2009). Absolute metabolite concentrations and implied enzyme active site occupancy in Escherichia coli. Nat. Chem. Biol..

[31] Milo R, Jorgensen P, Moran U, Weber G, Springer M (2010). Bionumbers-the database of key numbers in molecular and cell biology. Nucleic Acids Res..

[32] Siess EA, Kientsch-Engel RI, Wieland OH (1984). Concentration of free oxaloacetate in the mitochondrial compartment of isolated liver cells. Biochem. J..

[33] Psychogios N (2011). The human serum metabolome. PloS One.

[34] Ahn E, Kumar P, Mukha D, Tzur A, Shlomi T (2017). Temporal fluxomics reveals oscillations in TCA cycle flux throughout the mammalian cell cycle. Mol. Syst. Biol..

[35] Hoffmann G (1993). Physiology and pathophysiology of organic acids in cerebrospinal fluid. J. Inherit. Metab. Dis..

[36] Mogilevskaya E, Demin O, Goryanin I (2006). Kinetic model of mitochondrial Krebs cycle: Unraveling the mechanism of salicylate hepatotoxic effects. J. Biol. Phys..

[37] Kohlschütter A (1982). A familial progressive neurodegenerative disease with 2-oxoglutaric aciduria. Eur. J. Pediatr..

[38] Hansford RG, Johnson RN (1975). The steady state concentrations of coenzyme a-sh and coenzyme a thioester, citrate, and isocitrate during tricarboxylate cycle oxidations in rabbit heart mitochondria. J. Biol. Chem..

[39] Saltiel AR, Kahn CR (2001). Insulin signalling and the regulation of glucose and lipid metabolism. Nature.

[40] Guo J (2022). Aging and aging-related diseases: From molecular mechanisms to interventions and treatments. Signal Transduct. Target. Ther..

[41] Ahmad E, Lim S, Lamptey R, Webb DR, Davies MJ (2022). Type 2 diabetes. Lancet.

[42] Nonguierma E (2022). Improving obesogenic dietary behaviors among adolescents: A systematic review of randomized controlled trials. Nutrients.

[43] van Beek JH, Kirkwood TB, Bassingthwaighte JB (2016). Understanding the physiology of the ageing individual: Computational modelling of changes in metabolism and endurance. Interface Focus.

[44] Faubert B, Solmonson A, DeBerardinis RJ (2020). Metabolic reprogramming and cancer progression. Science.

[45] Warburg O (1925). The metabolism of carcinoma cells. J. Cancer Res..

[46] Johri A, Beal MF (2012). Mitochondrial dysfunction in neurodegenerative diseases. J. Pharmacol. Exp. Ther..

[47] Trifunovic A, Larsson N-G (2008). Mitochondrial dysfunction as a cause of ageing. J. Intern. Med..

[48] Hall KD (2006). Computational model of in vivo human energy metabolism during semistarvation and refeeding. Am. J. Physiol. Endocrinol. Metab..

[49] Rozendaal YJ, Wang Y, Hilbers PA, van Riel NA (2019). Computational modelling of energy balance in individuals with metabolic syndrome. BMC Syst. Biol..

[50] Shampine LF, Thompson S, Kierzenka J, Byrne G (2005). Non-negative solutions of odes. Appl. Math. Comput..

[51] Lee JM, Gianchandani EP, Papin JA (2006). Flux balance analysis in the era of metabolomics. Brief. Bioinform..

[52] Orth JD, Thiele I, Palsson BØ (2010). What is flux balance analysis?. Nat. Biotechnol..

[53] Raman K, Chandra N (2009). Flux balance analysis of biological systems: Applications and challenges. Brief. Bioinform..

[54] Massey WA (1985). Asymptotic analysis of the time dependent m/m/1 queue. Math. Oper. Res..

[55] Teusink B (2000). Can yeast glycolysis be understood in terms of in vitro kinetics of the constituent enzymes? Testing biochemistry. Eur. J. Biochem..

[56] Rossi RJ (2018). Mathematical Statistics: An Introduction to Likelihood Based Inference.

[57] Singh VK, Ghosh I (2006). Kinetic modeling of tricarboxylic acid cycle and glyoxylate bypass in Mycobacterium tuberculosis, and its application to assessment of drug targets. Theor. Biol. Med. Model..

