# Publisher Correction: Integrating glycolysis, citric acid cycle, pentose phosphate pathway, and fatty acid beta-oxidation into a single computational model

**DOI:** 10.1038/s41598-023-45724-w

**Published:** 2023-10-31

**Authors:** Sylwester M. Kloska, Krzysztof Pałczyński, Tomasz Marciniak, Tomasz Talaśka, Beata J. Wysocki, Paul Davis, Tadeusz A. Wysocki

**Affiliations:** 1 Faculty of Medicine, Nicolaus Copernicus University Ludwik Rydygier Collegium Medicum, 85-094 Bydgoszcz, Poland; 2 Faculty of Telecommunications, Computer Science and Electrical Engineering, Bydgoszcz University of Science and Technology, 85-796 Bydgoszcz, Poland; 3 Department of Biology, University of Nebraska at Omaha, Omaha, NE 68182 USA; 4 Department of Electrical and Computer Engineering, University of Nebraska-Lincoln, Omaha, NE 68182 USA

Correction to: *Scientific Reports* 10.1038/s41598-023-41765-3, published online 02 September 2023

The original version of this Article contained an error in the spelling of the author Tadeusz A. Wysocki, which was incorrectly given as Tadeusz A. Wysoki.

The original Article has been corrected.

