# Supplementary material for: Integrating glycolysis, citric acid cycle, pentose phosphate pathway, and fatty acid beta-oxidation into a single computational model
Source: Sci Rep. 2023 Sep 2;13:14484. doi: 10.1038/s41598-023-41765-3 (PMC10475038; doi:10.1038/s41598-023-41765-3)
Supplement: Supplementary file 1 — Supplementary Information. [file 41598_2023_41765_MOESM1_ESM.pdf]

Integrating glycolysis, citric acid cycle, pentose phosphate pathway, and fatty acid beta-oxidation into a single computational model

Sylwester M. Kloska[1,*], Krzysztof Pałczyński[2], Tomasz Marciniak[2], Tomasz Talaśka[2], Beata J. Wysocki[3], Paul H. Davis[3], and Tadeusz A. Wysocki[2,4]

[1]Department of Forensic Medicine, Nicolaus Copernicus University Ludwik Rydygier Collegium Medicum, 85-094 Bydgoszcz, Poland; [2]Faculty of Telecommunications, Computer Science and Electrical Engineering, Bydgoszcz University of Science and Technology, 85-796 Bydgoszcz, Poland; [3]Department of Biology, University of Nebraska at Omaha, Omaha, NE 68182, USA; [4]Department of Electrical and Computer Engineering, University of Nebraska-Lincoln, Omaha, NE 68182, USA.

Corresponding author e-mail: 503013@stud.umk.pl

Metabolites' and Energy Nucleotides concentration used/tested in the model

Table 1. Initial concentrations of glycolytic metabolites

Metabolite	Concentration [mmol/L]	Reference	Data source (cell line, tissue, organ, species, computational modeling)
Glucose (Glc)	5	1	Human erythrocytes
G6P	0.039	1	Human erythrocytes
F6P	0.013	2	Rat Liver Parenchymal Cells
F1,6BP	0.00231	1	Human erythrocytes
F2,6BP	0.004	2	Rat Liver Parenchymal Cells
DHAP	0.02	1	Human erythrocytes
G3P	0.00194	2	Rat Liver Parenchymal Cells
1,3BPG	0.000369	2	Rat Liver Parenchymal Cells
3PG	0.069	1	Human erythrocytes
2PG	0.01	2	Rat Liver Parenchymal Cells
PEP	0.017	1	Human erythrocytes
Pyruvate	0.077-0.085	3	Human erythrocytes
	5.88	4	---

	0.14	[5]	Computational modeling, based on 8 scientific positions
	0.0586	[2]	Rat Liver Parenchymal Cells

Table 2. Initial concentrations of the pentose phosphate pathway metabolites

Metabolite	Concentration [mmol/L]	Reference	Data source (cell line, tissue, organ, species, computational modeling)
G6P	0.0026	[6]	Rat liver cells
6-P-gluconolactone (PGL)	5×10^{-6}	[6]	Rat liver cells
6-P-gluconate (6PG)	0.018	[6]	Rat liver cells
Ribulose-5-P (Ru5P)	0.012	[6]	Rat liver cells
Ribose-5-P (R5P)	0.009	[6]	Rat liver cells
Xylulose-5-P (X5P)	0.018	[6]	Rat liver cells
Glyceraldehyde-3-P (G3P)	0.00234	[6]	Rat liver cells
	0.00194	[2]	Rat Liver Parenchymal Cells
Sedoheptulose-7-P (S7P)	0.068	[6]	Rat liver cells
Erythrose-4-P (E4P)	0.004	[6]	Rat liver cells
Fructose-6-P (F6P)	0.083	[6]	Rat liver cells
	0.013	[2]	Rat Liver Parenchymal Cells

Table 3. Initial concentrations of the fatty acid beta-oxidation metabolites

Metabolite	Concentration [mmol/L]	Reference	Data source (cell line, tissue, organ, species, computational modeling)
C16AcylCarCYT	0	[7]	Rat liver/computational modeling
C16AcylCarMAT	0	[7]	Rat liver/computational modeling
C16AcylCoAMAT	0	[7]	Rat liver/computational modeling
C16EnoylCoAMAT	0	[7]	Rat liver/computational modeling
C16HydroxyacylCoAMAT	0	[7]	Rat liver/computational modeling
C16KetoacylCoAMAT	0	[7]	Rat liver/computational modeling
C14AcylCarCYT	0	[7]	Rat liver/computational modeling
C14AcylCarMAT	0	[7]	Rat liver/computational modeling

C14AcylCoAMAT	0	[7]	Rat liver/computational modeling
C14EnoylCoAMAT	0	[7]	Rat liver/computational modeling
C14HydroxyacylCoAMAT	0	[7]	Rat liver/computational modeling
C14KetoacylCoAMAT	0	[7]	Rat liver/computational modeling
C12AcylCarCYT	0	[7]	Rat liver/computational modeling
C12AcylCarMAT	0	[7]	Rat liver/computational modeling
C12AcylCoAMAT	0	[7]	Rat liver/computational modeling
C12EnoylCoAMAT	0	[7]	Rat liver/computational modeling
C12HydroxyacylCoAMAT	0	[7]	Rat liver/computational modeling
C12KetoacylCoAMAT	0	[7]	Rat liver/computational modeling
C10AcylCarCYT	0	[7]	Rat liver/computational modeling
C10AcylCarMAT	0	[7]	Rat liver/computational modeling
C10AcylCoAMAT	0	[7]	Rat liver/computational modeling
C10EnoylCoAMAT	0	[7]	Rat liver/computational modeling
C10HydroxyacylCoAMAT	0	[7]	Rat liver/computational modeling
C10KetoacylCoAMAT	0	[7]	Rat liver/computational modeling
C8AcylCarCYT	0	[7]	Rat liver/computational modeling
C8AcylCarMAT	0	[7]	Rat liver/computational modeling
C8AcylCoAMAT	0	[7]	Rat liver/computational modeling
C8EnoylCoAMAT	0	[7]	Rat liver/computational modeling
C8HydroxyacylCoAMAT	0	[7]	Rat liver/computational modeling
C8KetoacylCoAMAT	0	[7]	Rat liver/computational modeling
C6AcylCarCYT	0	[7]	Rat liver/computational modeling
C6AcylCarMAT	0	[7]	Rat liver/computational modeling
C6AcylCoAMAT	0	[7]	Rat liver/computational modeling

C6EnoylCoAMAT	0	[7]	Rat liver/computational modeling
C6HydroxyacylCoAMAT	0	[7]	Rat liver/computational modeling
C6KetoacylCoAMAT	0	[7]	Rat liver/computational modeling
C4AcylCarCYT	0	[7]	Rat liver/computational modeling
C4AcylCarMAT	0	[7]	Rat liver/computational modeling
C4AcylCoAMAT	0	[7]	Rat liver/computational modeling
C4EnoylCoAMAT	0	[7]	Rat liver/computational modeling
C4HydroxyacylCoAMAT	0	[7]	Rat liver/computational modeling
C4AcetoacylCoAMAT	0	[7]	Rat liver/computational modeling
AcetylCoAMAT	70	[7]	Rat liver/computational modeling
FADHMAT	0.46	[7]	Rat liver/computational modeling
NADHMAT	16	[7]	Rat liver/computational modeling

Table 4. Initial concentrations of the citric acid cycle metabolites

Metabolite	Concentration [mmol/L]	Reference	Data source (cell line, tissue, organ, species, computational modeling)
Pyruvate	0.077-0.085	[3]	Human erythrocytes
	5.88	[4]	---
	0.14	[5]	Computational modeling, based on 8 scientific positions
	0.0586	[2]	Rat Liver Parenchymal Cells
Acetyl-CoA	0.0288	[4]	---
	0.61	[8]	Escherichia coli
	0.07	[5]	Computational modeling, based on 8 scientific positions
	0.5	[9]	---
Citrate	0.584	[4]	---
	2	[8]	Escherichia coli
	0.4	[5]	Computational modeling, based on 8 scientific positions
	0.19	[10]	HeLa cells
Cis-aconitate	0.0016	[8]	Escherichia coli

Isocitrate	0.0321	[4]	---
	0.02	[9]	---
α-ketoglutarate	0.797	[4]	---
	0.44	[8]	Escherichia coli
	0.25	[5]	Computational modeling, based on 8 scientific positions
	0.54	[11,12]	Rat liver
Succinyl-CoA	0.23	[8]	Escherichia coli
	0.0068	[4]	---
	0.36–0.91	[12,13]	Rabbit heart mitochondria
Succinate	0.57	[8]	Escherichia coli
	0.007	[12]	Computational modeling
	0.352	[9]	---
	0.07	[14]	---
Fumarate	0.485	[4]	---
	0.12	[8]	Escherichia coli
	0.124	[4]	Yeast
	1.94	[12]	Computational modeling
Malate	1.7	[8]	Escherichia coli
	1.39	[4]	---
	0.324-0.495	[12,15]	Guinea pig liver
	0.5-2.5	[12,16]	Rat liver
	0.87	[10]	HeLa cells
Oxaloacetate	0.00201	[4]	---
	0.002-0.006	[12,17]	Liver cells
	0.005	[5]	Computational modeling based on 7 scientific positions

Table 5. Additional Metabolites and Energy Nucleotides

Metabolite	Concentration [mmol/L]	Reference	Data source (cell line, tissue, organ, species, computational modeling)
Coenzyme A	0.16-0.79	[12,13]	Rabbit heart mitochondria
	1.4	[8]	Escherichia coli
	4.04	[4]	---
	0.044	[9]	---
Adenosine triphosphate (ATP)	3.23	[5]	Computational modeling based on 3 scientific positions
	4.67	[4]	---
	9.6	[8]	Escherichia coli
	2.52	[18]	Muscle tissue
	0.159	[1]	Human erythrocytes
Adenosine diphosphate (ADP)	0.56	[8]	Escherichia coli
	0.569	[4]	---
	$1.29x10^{-6}$	[18]	Muscle tissue
	0.0937	[1]	Human erythrocytes

Adenosine monophosphate (AMP)	0.08-0.4	[19]	Liver cells
	0.0423	[4]	---
	0.28	[8]	Escherichia coli
	2	[18]	Muscle tissue
	0.03	[1]	Human erythrocytes
Guanosine diphosphate (GDP)	0.0012	[9]	---
NAD^+	0.04-0.09	[3]	Human erythrocytes
	0.0599	[1]	Human erythrocytes
	0.502	[4]	---
	2.6	[8]	Escherichia coli
	0.94	[5]	Computational modeling based on 2 scientific positions
	1.55	[18]	Muscle tissue
	0.099	[9]	---
NADH	0.000245	[1]	Human erythrocytes
	0.075	[4]	---
	0.025	[9]	---
NADP+	0.001	[6]	Rat liver cells
NADPH	0.0002	[6]	Rat liver cells
H_2O	0.170	[9]	---
H^+	7.21×10^{-5}	[20]	Human liver cells
	5.2×10^{-6}	[12]	---
Inorganic phosphate (P_i)	2.44	[5,17]	Computational modeling
	1	[1]	Human erythrocytes
	0.05	[9]	---
Hydrogencarbonate (HCO^{3-})	0.003	[9]	---
MgATP	1.52	[1]	Human erythrocytes
MgADP	0.11	[1]	Human erythrocytes
Mg	0.4	[1]	Human erythrocytes
2,3BPG	3.1	[1]	Human erythrocytes
GSH	3.2	[1]	Human erythrocytes
ALA	0.2	[2]	Rat Liver Parenchymal Cells
G1,6BP	0.106	[1]	Human erythrocytes

Table 6. Kinetic constants used in the model

Kinetic constant	Value	Reference	Source
Reaction: Glucose → Glucose-6-phosphate			
$V_{F_{Hexokinase}}$	262.1443	[9]	---
K_a	0.14	[9]	---
K_b	1.00	[9]	---
K_p	0.02	[9]	---
K_q	3.5	[9]	---
K_{eq}	651	[9]	---
Reaction: Glucose-6-phosphate → Fructose-6-phosphate			
$V_{F_{Glucose-6-phosphate\ isomerase}}$	4.8×10^{-4} mmol/min	[9]	---

$V_{R_{Glucose-6-phosphate\ isomerase}}$	4*10^{-4} mmol/min	9	---
K_F	0.3 mM	9	---
K_R	0.123 mM	9	---
Reaction: Fructose-6-phosphate → Fructose 2,6-bisphosphate			
$V_{F_{PFK-2}}$	41.6 mmol/min	9	---
$K_{m_{ATP(PFK2)}}$	0.15 mM	9	---
$K_{m_{F6P(PFK2)}}$	0.032 mM	9	---
$K_{m_{F26BP(PFK2)}}$	0.008 mM	9	---
$K_{m_{ADP(PFK2)}}$	0.062 mM	9	---
$K_{i_{ATP(PFK2)}}$	0.15 mM	9	---
$K_{i_{F6P(PFK2)}}$	0.001 mM	9	---
$K_{i_{F26BP(PFK2)}}$	0.02 mM	9	---
$K_{i_{ADP(PFK2)}}$	0.23 mM	9	---
$K_{i_{PEP(PFK2)}}$	0.013 mM	9	---
$K_{eq(PFK2)}$	16 mM	9	---
Reaction: Fructose 2,6-bisphosphate → Fructose-6-phosphate			
$V_{F_{F26BPase}}$	416 mmol/min	9	---
$K_{m_{F26BP(F26BPase)}}$	0.001 mM	9	---
$K_{i_{F6P(F26BPase)}}$	0.0025 mM	9	---
Reaction: Fructose-6-phosphate → Fructose 1,6-bisphosphate			
$V_{F_{PFK-1}}$	15.5*10^{-2} mmol/min	9	---
$V_{R_{PFK-1}}$	0.678 mmol/min	9	---
$K_{F6P(PFK)}$	0.06 mM	9	---
$K_{MgATP(PFK)}$	0.068 mM	9	---
$K_{MgADP(PFK)}$	0.54 mM	9	---
$K_{F16BP(PFK)}$	0.65 mM	9	---
$K_{F26BP(PFK)}$	5.5*10^{-3} mM	9	---
$K_{G16BP(PFK)}$	0.1 mM	9	---
$K_{ATP(PFK)}$	0.1 mM	9	---
$K_{AMP(PFK)}$	0.3 mM	9	---
$K_{Mg(PFK)}$	0.2 mM	9	---
$K_{Pi(PFK)}$	30 mM	9	---
$K_{23BPG(PFK)}$	0.5 mM	9	---
L_{PFK}	0.002 mM	9	L_{PFK} represents the equilitbrium constant between the two states of the enzyme in the absence of any substrates.
Reaction: Fructose 1,6-bisphosphate → Dihydroxyacetone phosphate + Glyceraldehyde 3-phosphate			
$V_{m_{F(Fructose-bisphosphate\ aldolase)}}$	0.0675 mmol/min	9	---
$V_{m_{R(Fructose-bisphosphate\ aldolase)}}$	0.00232 mmol/min	9	---
$K_{F16BP(Aldo)}$	0.05 mM	9	---
$K_{i_{F16BP(Aldo)}}$	0.0198 mM	9	---

$K_{DHAP(Aldo)}$	0.035 mM	9	---
$K_{i_{DHAP(Aldo)}}$	0.011 mM	9	---
$K_{GP3(Aldo)}$	0.189 mM	9	---
$K_{i_{23BPG(Aldo)}}$	1.5 mM	9	---
Reaction: Dihydroxyacetone phosphate → Glyceraldehyde 3-phosphate			
$V_{F_{Triosephosphate\ isomerase}}$	0.051 mmol/min	9	---
$V_{R_{Triosephosphate\ isomerase}}$	0.461 mmol/min	9	---
$K_{F(Isomerase)}$	0.162 mM	9	---
$K_{R(Isomerase)}$	0.43 mM	9	---
Reaction: Glyceraldehyde 3-phosphate → 1,3-Bisphosphoglycerate			
$V_{mF_{GAPD}}$	$5.317*10^{-3}$ mmol/min	9	---
$V_{mR_{GAPD}}$	$3.919*10^{-3}$ mmol/min	9	---
$K_{NAD(GAPD)}$	0.045 mM	9	---
$K_{i_{NAD(GAPD)}}$	0.045 mM	9	---
$K_{Pi(GAPD)}$	3.16 mM	9	---
$K_{i_{Pi(GAPD)}}$	3.16 mM	9	---
$K_{G3P(GAPD)}$	0.095 mM	9	---
$K_{i_{G3P(GAPD)}}$	$1.59*10^{-16}$ mM	9	---
$K_{i_{G3P(GAPD')}}$	0.031 mM	9	---
$K_{NADH(GAPD)}$	0.033 mM	9	---
$K_{i_{NADH(GAPD)}}$	0.01 mM	9	---
$K_{13BPG(GAPD)}$	0.00671 mM	9	---
$K_{i_{13BPG(GAPD)}}$	$1.52*10^{-18}$ mM	9	---
$K_{i_{13BPG(GAPD')}}$	0.001 mM	9	---
$K_{eq(GAPD)}$	$1.9*10^{-8}$ mM	9	---
Reaction: 1,3-Bisphosphoglycerate → 3-Phosphoglycerate			
$V_{mF_{PGK}}$	$5.96*10^{4}$ mmol/min	9	---
$V_{mR_{PGK}}$	$2.39*10^{4}$ mmol/min	9	---
$K_{MgADP(PGK)}$	0.1 mM	9	---
$K_{i_{MgADP(PGK)}}$	0.08 mM	9	---
$K_{13BPG(PGK)}$	0.002 mM	9	---
$K_{i_{13BPG(PGK)}}$	1.6 mM	9	---
$K_{MgATP(PGK)}$	1.0 mM	9	---
$K_{i_{MgATP(PGK)}}$	0.186 mM	9	---
$K_{3PG(PGK)}$	1.1 mM	9	---
$K_{i_{3PG(PGK)}}$	0.205 mM	9	---
$K_{eq(PGK)}$	0.0032 mM	9	---
Reaction: 3-Phosphoglycerate → 2-Phosphoglycerate			
$V_{mF_{PGAM}}$	$4.894*10^{5}$ mmol/min	9	---
$V_{mR_{PGAM}}$	$4.395*10^{5}$ mmol/min	9	---
$K_{3PG(PGAM)}$	0.168 mM	9	---
$K_{2PG(PGAM)}$	0.0256 mM	9	---
$K_{eq(PGAM)}$	0.17 mM	9	---
Reaction: 2-Phosphoglycerate → Phosphoenolpyruvate			
$V_{mF_{Eno}}$	$2.106*10^{4}$ mmol/min	9	---

$V_{mR_{Eno}}$	5.542*10^{3} mmol/min	[9]	---
$K_{i_{Mg(Eno)}}$	0.14 mM	[9]	---
$K_{PEP(PGAM)}$	0.11 mM	[9]	---
$K_{2PG(Eno)}$	0.046 mM	[9]	---
$K_{eq(Eno)}$	3.0 mM	[9]	---
Reaction: Phosphoenolpyruvate → Pyruvate			
$V_{mF_{PK}}$	2.02*10^{4} mmol/min	[9]	---
$V_{mR_{PK}}$	47.5	[9]	---
$K_{PEP(PK)}$	0.225 mM	[9]	---
$K_{MgADP(PK)}$	0.474 mM	[9]	---
$K_{MgATP(PK)}$	3.0 mM	[9]	---
$K_{ATP(PK)}$	3.39 mM	[9]	---
$K_{PYR(PK)}$	4.0 mM	[9]	---
$K_{F16BP(PK)}$	0.04 mM	[9]	---
$K_{G16BP(PK)}$	0.1 mM	[9]	---
$K_{ALA(PK)}$	0.02 mM	[9]	---
L_{PK}	0.398 mM	[9]	L_{PK} represents the equilitbrium constant between the two states of the enzyme in the absence of any substrates.
Reaction: Glucose-6-phosphate → 6-P-gluconolactone			
V_1	5.9*10^{-6} mmol/min	[21]	Pig liver cells
$K_{(NADP)}$	4.8*10^{-3} mM	[21]	Pig liver cells
$K_{(G6P)}$	3.6*10^{-2} mM	[21]	Pig liver cells
$K_{i(NADP)}$	9*10^{-3} mM	[21]	Pig liver cells
$K_{i(NADPH)}$	1.1*10^{-3} mM	[21]	Pig liver cells
Reaction: 6-P-gluconolactone → gluconate-6-phosphate			
V_{2F}	5.9*10^{-6} mmol/min	[6]	---
V_{2R}	1.232*10^{-9} mmol/min	[6]	---
$K_{(PGL)}$	8*10^{-2} mM	[6]	---
$K_{(6PG)}$	8*10^{-2} mM	[6]	---
Reaction: gluconate-6-phosphate → ribulose-5-phosphate			
V_{3F}	4.93*10^{-6} mmol/min	[6]	---
V_{3R}	1.064*10^{-13} mmol/min	[6]	---
$K_{(NADP)}$	1.35*10^{-2} mM	[6]	---
$K_{i(NADP)}$	4.8*10^{-3} mM	[6]	---
$K_{i(NADPH)}$	5.1*10^{-3} mM	[6]	---
$K_{(6PG)}$	2.92*10^{-2} mM	[6]	---
$K_{(CO_2)}$	34 mM	[6]	---
$K_{(Ru5P)}$	2*10^{-2} mM	[6]	---
$K_{(NADPH)}$	2.2*10^{-4} mM	[6]	---

$K_{(eq)}$	66 mM	6	---
$K_{i(6PG)}$	2.176 mM	6	---
$K_{i(CO_2)}$	1.387*10^{-2} mM	6	---
$K_{i(Ru5P)}$	4.488*10^{-8} mM	6	---
Reaction: ribulose-5-phosphate → ribose-5-phosphate			
V_{4AF}	5.9*10^{-6} mmol/min	6	---
V_{4AR}	1.1225*10^{-5} mmol/min	6	---
$K_{(Ru5P)}$	7.8*10^{-1} mM	6	---
$K_{(R5P)}$	2.2 mM	6	---
Reaction: ribulose-5-phosphate → xylulose-5-phosphate			
V_{4BF}	5.9*10^{-6} mmol/min	6	---
V_{4BR}	8.48*10^{-6} mmol/min	6	---
$K_{(Ru5P)}$	0.19 mM	6	---
$K_{(X5P)}$	0.5 mM	6	---
Reaction: ribose-5-phosphate + xylulose-5-phosphate → glyceraldehyde 3-phosphate + sedoheptulose-7-phosphate & Reaction: xylulose-5-phosphate + erythrose-4-phosphate → fructose-6-phosphate + glyceraldehyde 3-phosphate			
K_1	6*10^{-4} mM	6	---
K_2	1.1*10^{-9} mM	6	---
K_3	1.006*10^{-5} mM	6	---
K_4	9.9*10^{-10} mM	6	---
K_5	1.09 mM	6	---
K_6	3.2*10^{-3} mM	6	---
K_7	15.5 mM	6	---
K_8	3.8*10^{-1} mM	6	---
K_9	1.548*10^{-3} mM	6	---
K_{10}	3.8*10^{-1} mM	6	---
K_{11}	1267 mM	6	---
K_{12}	6050 mM	6	---
K_{13}	1*10^{-2} mM	6	---
K_{14}	1000 mM	6	---
K_{15}	1*10^{-2} mM	6	---
K_{16}	8.6 mM	6	---
K_{17}	1000 mM	6	---
K_{18}	86400 mM	6	---
K_{19}	8640 mM	6	---
K_{20}	5.9*10^{-6} mM	6	---
K_{21}	2.2*10^{-9} mM	6	---
K_{22}	3.802*10^{-7} mM	6	---
K_{23}	5.9*10^{-10} mmol	6	---
$K_{i(R5P)}$	0.82 mM	6	---
$K_{i(X5P)}$	3.6 mM	6	---
Reaction: glyceraldehyde 3-phosphate + sedoheptulose-7-phosphate → erythrose-4-phosphate + fructose-6-phosphate			
V_{7F}	5.9*10^{-6} mmol/min	6	---

V_{7R}	1.776*10^{-6} mmol/min	[6]	---
$K_{(S7P)}$	1.8*10^{-1} mM	[6]	---
$K_{(G3P)}$	2.2*10^{-1} mM	[6]	---
$K_{(E4P)}$	7*10^{-3} mM	[6]	---
$K_{(F6P)}$	2*10^{-1} mM	[6]	---
$K_{i(S7P)}$	1.8*10^{-1} mM	[6]	---
$K_{i(E4P)}$	7*10^{-3} mM	[6]	---
$K_{i(F6P)}$	2*10^{-1} mM	[6]	---
Reaction: $C16AcylCoACYT \rightarrow C16AcylCarCYT$			
v_{cat1}	$0.012\ [\frac{\mu mol}{min * mgProtein}]$	[7]	Rat liver/computational modeling
$K_{M_{C16AcylCoACYT}}$	$13.8\ [\mu M]$	[7]	Rat liver/computational modeling
$K_{M_{CarCYT}}$	$250\ [\mu M]$	[7]	Rat liver/computational modeling
$K_{M_{C16AcylCarCYT}}$	$136\ [\mu M]$	[7]	Rat liver/computational modeling
$K_{M_{CoACYT}}$	$40.7\ [\mu M]$	[7]	Rat liver/computational modeling
Reaction: $C16AcylCarCYT \rightarrow C16AcylCarMAT$			
v_{cact}	$0.42\ [\frac{\mu mol}{min * mgProtein}]$	[7]	Rat liver/computational modeling
$K_{M_{C16AcylCarCYT}}$	$15\ [\mu M]$	[7]	Rat liver/computational modeling
$K_{M_{CarMAT}}$	$130\ [\mu M]$	[7]	Rat liver/computational modeling
$K_{M_{C16AcylCarMAT}}$	$15\ [\mu M]$	[7]	Rat liver/computational modeling
$K_{M_{CarCYT}}$	$130\ [\mu M]$	[7]	Rat liver/computational modeling
Reaction: $C16AcylCarMAT \rightarrow C16AcylCoAMAT$			
v_{cat2}	$0.391\ [\frac{\mu mol}{min * mgProtein}]$	[7]	Rat liver/computational modeling
$K_{M_{C16AcylCarMAT}}$	$51\ [\mu M]$	[7]	Rat liver/computational modeling

$K_{M_{CoAMAT}}$	$30\ [\mu M]$	[7]	Rat liver/computational modeling
$K_{M_{C16AcylCoAMAT}}$	$38\ [\mu M]$	[7]	Rat liver/computational modeling
$K_{M_{CarMAT}}$	$350\ [\mu M]$	[7]	Rat liver/computational modeling
Reaction: $C16AcylCoAMAT \rightarrow C16EnoylCoAMAT$			
v_{vlcad}	$0.008\ [\frac{\mu mol}{min * mgProtein}]$	[7]	Rat liver/computational modeling
$K_{M_{C16AcylCoAMAT}}$	$6.5\ [\mu M]$	[7]	Rat liver/computational modeling
$K_{M_{C16EnoylCoAMAT}}$	$1.08\ [\mu M]$	[7]	Rat liver/computational modeling
Reaction: $C16EnoylCoAMAT \rightarrow C16HydroxyacylCoAMAT$			
v_{crot}	$3.6\ [\frac{\mu mol}{min * mgProtein}]$	[7]	Rat liver/computational modeling
$K_{M_{C16EnoylCoAMAT}}$	$150\ [\mu M]$	[7]	Rat liver/computational modeling
$K_{M_{C16HydroxyacylCoAMAT}}$	$40\ [\mu M]$	[7]	Rat liver/computational modeling
Reaction: $C16HydroxyacylCoAMAT \rightarrow C16KetoacylCoAMAT$			
v_{mschad}	$1\ [\frac{\mu mol}{min * mgProtein}]$	[7]	Rat liver/computational modeling
$K_{M_{C16HydroxyacylCoAMAT}}$	$1.5\ [\mu M]$	[7]	Rat liver/computational modeling
$K_{M_{C16KetoacylCoAMAT}}$	$1.4\ [\mu M]$	[7]	Rat liver/computational modeling
Reaction: $C16KetoacylCoAMAT \rightarrow C14AcylCoAMAT + AcetylCoAMAT$			
v_{mckat}	$0.377\ [\frac{\mu mol}{min * mgProtein}]$	[7]	Rat liver/computational modeling
$K_{M_{C16KetoacylCoAMAT}}$	$1.1\ [\mu M]$	[7]	Rat liver/computational modeling
$K_{M_{CoAMAT}}$	$26.6\ [\mu M]$	[7]	Rat liver/computational modeling

$K_{M_{C14AcylCoAMAT}}$	$13.83\ [\mu M]$	[7]	Rat liver/computational modeling
$K_{M_{AcetylCoAMAT}}$	$30\ [\mu M]$	[7]	Rat liver/computational modeling
Reaction: $C14AcylCoAMAT \rightarrow C14EnoylCoAMAT$			
v_{vlcad}	$0.008\ [\frac{\mu mol}{min * mgProtein}]$	[7]	Rat liver/computational modeling
$K_{M_{C14AcylCoAMAT}}$	$4\ [\mu M]$	[7]	Rat liver/computational modeling
$K_{M_{C14EnoylCoAMAT}}$	$1.08\ [\mu M]$	[7]	Rat liver/computational modeling
Reaction: $C14EnoylCoAMAT \rightarrow C14HydroxyacylCoAMAT$			
v_{crot}	$3.6\ [\frac{\mu mol}{min * mgProtein}]$	[7]	Rat liver/computational modeling
$K_{M_{C14EnoylCoAMAT}}$	$100\ [\mu M]$	[7]	Rat liver/computational modeling
$K_{M_{C14HydroxyacylCoAMAT}}$	$45\ [\mu M]$	[7]	Rat liver/computational modeling
Reaction: $C14HydroxyacylCoAMAT \rightarrow C14KetoacylCoAMAT$			
v_{mschad}	$1\ [\frac{\mu mol}{min * mgProtein}]$	[7]	Rat liver/computational modeling
$K_{M_{C14HydroxyacylCoAMAT}}$	$1.8\ [\mu M]$	[7]	Rat liver/computational modeling
$K_{M_{C14KetoacylCoAMAT}}$	$1.4\ [\mu M]$	[7]	Rat liver/computational modeling
Reaction: $C14KetoacylCoAMAT \rightarrow C12AcylCoAMAT + AcetylCoAMAT$			
v_{mckat}	$0.377\ [\frac{\mu mol}{min * mgProtein}]$	[7]	Rat liver/computational modeling
$K_{M_{C14KetoacylCoAMAT}}$	$1.2\ [\mu M]$	[7]	Rat liver/computational modeling
$K_{M_{CoAMAT}}$	$26.6\ [\mu M]$	[7]	Rat liver/computational modeling
$K_{M_{C12AcylCoAMAT}}$	$13.83\ [\mu M]$	[7]	Rat liver/computational modeling

$K_{M_{AcetylCoAMAT}}$	$30\ [\mu M]$	[7]	Rat liver/computational modeling
Reaction: $C12AcylCoAMAT \rightarrow C12EnoylCoAMAT$			
v_{vlcad}	$0.008\ [\frac{\mu mol}{min * mgProtein}]$	[7]	Rat liver/computational modeling
$K_{M_{C12AcylCoAMAT}}$	$2.7\ [\mu M]$	[7]	Rat liver/computational modeling
$K_{M_{C12EnoylCoAMAT}}$	$1.08\ [\mu M]$	[7]	Rat liver/computational modeling
Reaction: $C12EnoylCoAMAT \rightarrow C12HydroxyacylCoAMAT$			
v_{crot}	$3.6\ [\frac{\mu mol}{min * mgProtein}]$	[7]	Rat liver/computational modeling
$K_{M_{C12EnoylCoAMAT}}$	$25\ [\mu M]$	[7]	Rat liver/computational modeling
$K_{M_{C12HydroxyacylCoAMAT}}$	$45\ [\mu M]$	[7]	Rat liver/computational modeling
Reaction: $C12HydroxyacylCoAMAT \rightarrow C12KetoacylCoAMAT$			
v_{mschad}	$1\ [\frac{\mu mol}{min * mgProtein}]$	[7]	Rat liver/computational modeling
$K_{M_{C12HydroxyacylCoAMAT}}$	$3.7\ [\mu M]$	[7]	Rat liver/computational modeling
$K_{M_{C12KetoacylCoAMAT}}$	$1.6\ [\mu M]$	[7]	Rat liver/computational modeling
Reaction: $C12KetoacylCoAMAT \rightarrow C10AcylCoAMAT + AcetylCoAMAT$			
v_{mckat}	$0.377\ [\frac{\mu mol}{min * mgProtein}]$	[7]	Rat liver/computational modeling
$K_{M_{C12KetoacylCoAMAT}}$	$1.3\ [\mu M]$	[7]	Rat liver/computational modeling
$K_{M_{CoAMAT}}$	$26.6\ [\mu M]$	[7]	Rat liver/computational modeling
$K_{M_{C10AcylCoAMAT}}$	$13.83\ [\mu M]$	[7]	Rat liver/computational modeling
$K_{M_{AcetylCoAMAT}}$	$30\ [\mu M]$	[7]	Rat liver/computational modeling
Reaction: $C10AcylCoAMAT \rightarrow C10EnoylCoAMAT$			

v_{lcad}	$0.01\ [\frac{\mu mol}{min * mgProtein}]$	[7]	Rat liver/computational modeling
$K_{M_{C10AcylCoAMAT}}$	$24.3\ [\mu M]$	[7]	Rat liver/computational modeling
$K_{M_{C10EnoylCoAMAT}}$	$1.08\ [\mu M]$	[7]	Rat liver/computational modeling
Reaction: $C10EnoylCoAMAT \rightarrow C10HydroxyacylCoAMAT$			
v_{crot}	$3.6\ [\frac{\mu mol}{min * mgProtein}]$	[7]	Rat liver/computational modeling
$K_{M_{C10EnoylCoAMAT}}$	$25\ [\mu M]$	[7]	Rat liver/computational modeling
$K_{M_{C10HydroxyacylCoAMAT}}$	$45\ [\mu M]$	[7]	Rat liver/computational modeling
Reaction: $C10HydroxyacylCoAMAT \rightarrow C10KetoacylCoAMAT$			
v_{mschad}	$1\ [\frac{\mu mol}{min * mgProtein}]$	[7]	Rat liver/computational modeling
$K_{M_{C10HydroxyacylCoAMAT}}$	$8.8\ [\mu M]$	[7]	Rat liver/computational modeling
$K_{M_{C10KetoacylCoAMAT}}$	$2.3\ [\mu M]$	[7]	Rat liver/computational modeling
Reaction: $C10KetoacylCoAMAT \rightarrow C8AcylCoAMAT + AcetylCoAMAT$			
v_{mckat}	$0.377\ [\frac{\mu mol}{min * mgProtein}]$	[7]	Rat liver/computational modeling
$K_{M_{C10KetoacylCoAMAT}}$	$2.1\ [\mu M]$	[7]	Rat liver/computational modeling
$K_{M_{CoAMAT}}$	$26.6\ [\mu M]$	[7]	Rat liver/computational modeling
$K_{M_{C8AcylCoAMAT}}$	$13.83\ [\mu M]$	[7]	Rat liver/computational modeling
$K_{M_{AcetylCoAMAT}}$	$30\ [\mu M]$	[7]	Rat liver/computational modeling
Reaction: $C8AcylCoAMAT \rightarrow C8EnoylCoAMAT$			
v_{lcad}	$0.01\ [\frac{\mu mol}{min * mgProtein}]$	[7]	Rat liver/computational modeling

$K_{M_{C8AcylCoAMAT}}$	$123\ [\mu M]$	[7]	Rat liver/computational modeling
$K_{M_{C8EnoylCoAMAT}}$	$1.08\ [\mu M]$	[7]	Rat liver/computational modeling
Reaction: $C8EnoylCoAMAT \rightarrow C8HydroxyacylCoAMAT$			
v_{crot}	$3.6\ [\frac{\mu mol}{min * mgProtein}]$	[7]	Rat liver/computational modeling
$K_{M_{C8EnoylCoAMAT}}$	$25\ [\mu M]$	[7]	Rat liver/computational modeling
$K_{M_{C8HydroxyacylCoAMAT}}$	$45\ [\mu M]$	[7]	Rat liver/computational modeling
Reaction: $C8HydroxyacylCoAMAT \rightarrow C8KetoacylCoAMAT$			
v_{mschad}	$1\ [\frac{\mu mol}{min * mgProtein}]$	[7]	Rat liver/computational modeling
$K_{M_{C8HydroxyacylCoAMAT}}$	$16.3\ [\mu M]$	[7]	Rat liver/computational modeling
$K_{M_{C8KetoacylCoAMAT}}$	$4.1\ [\mu M]$	[7]	Rat liver/computational modeling
Reaction: $C8KetoacylCoAMAT \rightarrow C6AcylCoAMAT + AcetylCoAMAT$			
v_{mckat}	$0.377\ [\frac{\mu mol}{min * mgProtein}]$	[7]	Rat liver/computational modeling
$K_{M_{C8KetoacylCoAMAT}}$	$3.2\ [\mu M]$	[7]	Rat liver/computational modeling
$K_{M_{CoAMAT}}$	$26.6\ [\mu M]$	[7]	Rat liver/computational modeling
$K_{M_{C6AcylCoAMAT}}$	$13.83\ [\mu M]$	[7]	Rat liver/computational modeling
$K_{M_{AcetylCoAMAT}}$	$30\ [\mu M]$	[7]	Rat liver/computational modeling
Reaction: $C6AcylCoAMAT \rightarrow C6EnoylCoAMAT$			
v_{mcad}	$0.081\ [\frac{\mu mol}{min * mgProtein}]$	[7]	Rat liver/computational modeling
$K_{M_{C6AcylCoAMAT}}$	$9.4\ [\mu M]$	[7]	Rat liver/computational modeling

$K_{M_{C6EnoylCoAMAT}}$	$1.08\ [\mu M]$	[7]	Rat liver/computational modeling
Reaction: $C6EnoylCoAMAT \rightarrow C6HydroxyacylCoAMAT$			
v_{crot}	$3.6\ [\frac{\mu mol}{min * mgProtein}]$	[7]	Rat liver/computational modeling
$K_{M_{C6EnoylCoAMAT}}$	$25\ [\mu M]$	[7]	Rat liver/computational modeling
$K_{M_{C6HydroxyacylCoAMAT}}$	$45\ [\mu M]$	[7]	Rat liver/computational modeling
Reaction: $C6HydroxyacylCoAMAT \rightarrow C6KetoacylCoAMAT$			
v_{mschad}	$1\ [\frac{\mu mol}{min * mgProtein}]$	[7]	Rat liver/computational modeling
$K_{M_{C6HydroxyacylCoAMAT}}$	$28.6\ [\mu M]$	[7]	Rat liver/computational modeling
$K_{M_{C6KetoacylCoAMAT}}$	$5.8\ [\mu M]$	[7]	Rat liver/computational modeling
Reaction: $C6KetoacylCoAMAT \rightarrow C4AcylCoAMAT + AcetylCoAMAT$			
v_{mckat}	$0.377\ [\frac{\mu mol}{min * mgProtein}]$	[7]	Rat liver/computational modeling
$K_{M_{C6KetoacylCoAMAT}}$	$6.7\ [\mu M]$	[7]	Rat liver/computational modeling
$K_{M_{CoAMAT}}$	$26.6\ [\mu M]$	[7]	Rat liver/computational modeling
$K_{M_{C4AcylCoAMAT}}$	$13.83\ [\mu M]$	[7]	Rat liver/computational modeling
$K_{M_{AcetylCoAMAT}}$	$30\ [\mu M]$	[7]	Rat liver/computational modeling
Reaction: $C4AcylCoAMAT \rightarrow C4EnoylCoAMAT$			
v_{mcad}	$0.081\ [\frac{\mu mol}{min * mgProtein}]$	[7]	Rat liver/computational modeling
$K_{M_{C4AcylCoAMAT}}$	$135\ [\mu M]$	[7]	Rat liver/computational modeling
$K_{M_{C4EnoylCoAMAT}}$	$1.08\ [\mu M]$	[7]	Rat liver/computational modeling
Reaction: $C4EnoylCoAMAT \rightarrow C4HydroxyacylCoAMAT$			

v_{crot}	$3.6\ [\frac{\mu mol}{min * mgProtein}]$	[7]	Rat liver/computational modeling
$K_{M_{C4EnoylCoAMAT}}$	$40\ [\mu M]$	[7]	Rat liver/computational modeling
$K_{M_{C4HydroxyacylCoAMAT}}$	$45\ [\mu M]$	[7]	Rat liver/computational modeling
Reaction: $C4HydroxyacylCoAMAT \rightarrow C4AcetoacetylCoAMAT$			
v_{mschad}	$1\ [\frac{\mu mol}{min * mgProtein}]$	[7]	Rat liver/computational modeling
$K_{M_{C4HydroxyacylCoAMAT}}$	$69.9\ [\mu M]$	[7]	Rat liver/computational modeling
$K_{M_{C4AcetoacetylCoAMAT}}$	$16.9\ [\mu M]$	[7]	Rat liver/computational modeling
Reaction: $C4AcetoacetylCoAMAT \rightarrow AcetylCoAMAT$			
v_{mckat}	$0.377\ [\frac{\mu mol}{min * mgProtein}]$	[7]	Rat liver/computational modeling
$K_{M_{C4AcetoacetylCoAMAT}}$	$12.4\ [\mu M]$	[7]	Rat liver/computational modeling
$K_{M_{CoAMAT}}$	$26.6\ [\mu M]$	[7]	Rat liver/computational modeling
$K_{M_{AcetylCoAMAT}}$	$30\ [\mu M]$	[7]	Rat liver/computational modeling
Reaction: Pyruvate → Acetyl-CoA			
$V_{F_{Pyruvate\ dehydrogenase}}$	0.077 mmol/min	[9]	Escherichia coli
$K_{S_{Pyruvate}}$	0.01mM	[9]	Escherichia coli
$K_{P_{Acetyl-CoA}}$	0.1mM	[9]	Escherichia coli
Reaction: Pyruvate → Oxaloacetate			
$V_{F_{Pyruvate\ carboxylase}}$	0.02 mmol/min	[9]	Escherichia coli
$K_{S_{Pyruvate}}$	0.22 mM	[9]	Escherichia coli
$K_{S_{HCO3-}}$	1.75 mM	[9]	Escherichia coli
$K_{S_{ATP}}$	0.08 mM	[9]	Escherichia coli
$K_{P_{Oxaloacetate}}$	2.2 mM	[9]	Escherichia coli
$K_{P_{Pi}}$	17.5 mM	[9]	Escherichia coli
$K_{P_{ADP}}$	0.8 mM	[9]	Escherichia coli
Reaction: Oxaloacetate → Citrate			
$V_{F_{Citrate\ synthase}}$	91.2 mmol/min	[22]	Escherichia coli
$K_{S_{Oxaloacetate}}$	0.03 mM	[22]	Escherichia coli
$K_{S_{Acetyl-CoA}}$	0.07 mM	[22]	Escherichia coli
$K_{P_{Citrate}}$	0.3 mM	[22]	Escherichia coli

$K_{Coenzyme\ A}$	0.7 mM	[22]	Escherichia coli
Reaction: Citrate → Cis-Aconitate			
$V_{F_{Aconitase}}$	91.2 mmol/min	[22]	Escherichia coli
$K_{S_{Citrate}}$	1.7 mM	[22]	Escherichia coli
$K_{P_{Cis-Aconitate}}$	3.33mM	[22]	Escherichia coli
Reaction: Cis-Aconitate → Isocitrate			
$V_{F_{Aconitase}}$	91.2 mmol/min	[22]	Escherichia coli
$K_{S_{Cis-Aconitate}}$	1.7 mM	[22]	Escherichia coli
$K_{P_{Isocitrate}}$	3.33mM	[22]	Escherichia coli
Reaction: Isocitrate → α-Ketoglutarate			
$V_{F_{isocitrate\ dehydrogenase}}$	14.72 mmol/min	[22]	Escherichia coli
$K_{S_{Isocitrate}}$	0.008 mM	[22]	Escherichia coli
$K_{P_{\alpha-Ketoglutarate}}$	0.13 mM	[22]	Escherichia coli
Reaction: α-Ketoglutarate → Succinyl-CoA			
$V_{F_{Ketoglutarate\ Dehydrogenase}}$	3.5 mmol/min	[22]	Escherichia coli
$K_{S_{P_{\alpha-Ketoglutarate}}}$	0.1 mM	[22]	Escherichia coli
$K_{P_{Succinyl-CoA}}$	1.0 mM	[22]	Escherichia coli
Reaction: Succinyl-CoA → Succinate			
$V_{F_{succinate\ thiokinase}}$	3.5mmol/min	[22]	Escherichia coli
$K_{S_{Succinyl-CoA}}$	0.2 mM	[22]	Escherichia coli
$K_{P_{Succinate}}$	5 mM	[22]	Escherichia coli
Reaction: Succinate → Fumarate			
$V_{F_{succinate\ dehydrogenase}}$	7.38 mmol/min	[22]	Escherichia coli
$K_{S_{Succinate}}$	0.02 mM	[22]	Escherichia coli
$K_{P_{Fumarate}}$	0.4 mM	[22]	Escherichia coli
Reaction: Fumarate → Malate			
$V_{F_{Fumarase}}$	44.64 mmol/min	[22]	Escherichia coli
$K_{S_{Fumarate}}$	0.15 mM	[22]	Escherichia coli
$K_{P_{Malate}}$	0.04 mM	[22]	Escherichia coli
Reaction: Malate → Oxaloacetate			
$V_{F_{malate\ dehydrogenase}}$	356.64mmol/min	[22]	Escherichia coli
$K_{S_{Malate}}$	2.6mM	[22]	Escherichia coli
$K_{P_{Oxaloacetate}}$	0.04 mM	[22]	Escherichia coli

References

1. Mulquiney, P. J. & Kuchel, P. W. Model of 2,3-bisphosphoglycerate metabolism in the human erythrocyte based on detailed enzyme kinetic equations: equations and parameter refinement. *Biochem. J.* **342**, 597–604 (1999).
2. Groen, A. K., Sips, H. J., Vervoorn, R. C. & Tager, J. M. Intracellular Compartment ation and Control of Alanine Metabolism in Rat Liver Parenchymal Cells. *Eur. J. Biochem.* **122**, 87–93 (1982).
3. Rapoport, T. A., Heinrich, R., Jacobasch, G. & Rapoport, S. A Linear Steady-State Treatment of Enzymatic Chains. *Eur. J. Biochem.* **42**, 107–120 (1974).
4. Park, J. O. *et al.* Metabolite concentrations, fluxes and free energies imply efficient enzyme usage. *Nat. Chem. Biol.* **12**, 1–15 (2016).
5. Nazaret, C., Heiske, M., Thurley, K. & Mazat, J. P. Mitochondrial energetic metabolism: A

simplified model of TCA cycle with ATP production. *J. Theor. Biol.* **258**, 455–464 (2009).
6. Sabate, L., Franco, R., Canela, E. I., Centelles, J. J. & Cascante, M. A model of the pentose phosphate pathway in rat liver cells. *Mol. Cell. Biochem.* **142**, 9–17 (1995).
7. van Eunen, K. *et al.* Biochemical Competition Makes Fatty-Acid β-Oxidation Vulnerable to Substrate Overload. *PLoS Comput. Biol.* **9**, 2–9 (2013).
8. Bennett, B. D. *et al.* Absolute metabolite concentrations and implied enzyme active site occupancy in Escherichia coli. *Nat. Chem. Biol.* **5**, 593–599 (2009).
9. Milo, R., Jorgensen, P., Moran, U., Weber, G. & Springer, M. BioNumbers--The database of key numbers in molecular and cell biology. *Nucleic Acids Res.* **38(Databas**, D750–D753 (2010).
10. Ahn, E., Kumar, P., Mukha, D., Tzur, A. & Shlomi, T. Temporal fluxomics reveals oscillations in TCA cycle flux throughout the mammalian cell cycle. *Mol. Syst. Biol.* **13**, 953 (2017).
11. Hoek, J. B., Ernster, L., De Haan, E. J. & Tager, J. M. The nicotinamide nucleotide specificity of glutamate dehydrogenase in intact RAT-liver mitochondria. *Biochim. Biophys. Acta - Bioenerg.* **333**, 546–559 (1974).
12. Mogilevskaya, E., Demin, O. & Goryanin, I. Kinetic model of mitochondrial Krebs cycle: Unraveling the mechanism of salicylate hepatotoxic effects. *J. Biol. Phys.* **32**, 245–271 (2006).
13. Hansford, R. G. & Johnson, R. N. The steady state concentrations of coenzyme A-SH and coenzyme A thioester, citrate, and isocitrate during tricarboxylate cycle oxidations in rabbit heart mitochondria. *J. Biol. Chem.* **250**, 8361–8375 (1975).
14. Albe, K. R., Butler, M. H. & Wright, B. E. Cellular concentrations of enzymes and their substrates. *J. Theor. Biol.* **143(2)**, 163–95 (1990).
15. Garber, A. J. & Hanson, R. W. The Interrelationships of the Various Pathways Forming Gluconeogenic Precursors in Guinea Pig Liver Mitochondria: THE INFLUENCE OF THE OXIDATION-REDUCTION STATE OF NICOTINAMIDE ADENINE DINUCLEOTIDES ON PHOSPHOENOLPYRUVATE, MALATE, AND ASPARTATE FORMATION. *J. Biol. Chem.* **246**, 589–598 (1971).
16. Williamson, D. H., Lund, P. & Krebs, H. A. The redox state of free nicotinamide-adenine dinucleotide in the cytoplasm and mitochondria of rat liver. *Biochem. J.* **103**, 514–527 (1967).
17. Siess, E. A., Kientsch-Engel, R. I. & Wieland, O. H. Concentration of free oxaloacetate in the mitochondrial compartment of isolated liver cells. *Biochem. J.* **218**, 171–176 (1984).
18. McCullough, M. B. A. & Wesley, R. A. In Silico Modelling of Human Energy Metabolism. *EC Microbiol.* **1**, 26–36 (2017).
19. Van Den Berghe, G., Bronfman, M., Vanneste, R. & Hers, H. G. The mechanism of adenosine triphosphate depletion in the liver after a load of fructose. A kinetic study of liver adenylate deaminase. *Biochem. J.* **162**, 601–609 (1977).
20. König, M., Bulik, S. & Holzhütter, H.-G. Quantifying the contribution of the liver to glucose homeostasis: a detailed kinetic model of human hepatic glucose metabolism. *PLoS Comput. Biol.* **8**, e1002577 (2012).
21. Kanji, M. I., Toews, M. L. & Carper, W. R. A kinetic study of glucose-6-phosphate dehydrogenase. *J. Biol. Chem.* **251**, 2258–2262 (1976).
22. Singh, D. & Pinjala, R. K. Kinetic modeling of tricarboxylic acid cycle and glyoxylate bypass in Mycobacterium tuberculosis, and its application to assessment of drug targets. *Vasa - J. Vasc. Dis.* **35**, 27–29 (2006).
